# Intercellular interaction between FAP^+^ fibroblasts and CD150^+^ inflammatory monocytes mediates fibrostenosis in Crohn’s disease

**DOI:** 10.1172/JCI173835

**Published:** 2024-07-23

**Authors:** Bo-Jun Ke, Saeed Abdurahiman, Francesca Biscu, Gaia Zanella, Gabriele Dragoni, Sneha Santhosh, Veronica De Simone, Anissa Zouzaf, Lies van Baarle, Michelle Stakenborg, Veronika Bosáková, Yentl Van Rymenant, Emile Verhulst, Sare Verstockt, Elliott Klein, Gabriele Bislenghi, Albert Wolthuis, Jan Frič, Christine Breynaert, Andre D’Hoore, Pieter Van der Veken, Ingrid De Meester, Sara Lovisa, Lukas J.A.C. Hawinkels, Bram Verstockt, Gert De Hertogh, Séverine Vermeire, Gianluca Matteoli

**Affiliations:** 1 Translational Research Center for Gastrointestinal Disorders (TARGID), Department of Chronic Diseases and Metabolism, KU Leuven, Leuven, Belgium.; 2 Centre for Inflammation Research, University of Edinburgh, Edinburgh, United Kingdom.; 3 Gastroenterology Research Unit, Department of Experimental and Clinical Biomedical Sciences, University of Florence, Florence, Italy.; 4 Department of Biology, Faculty of Medicine, Masaryk University, Brno, Czech Republic.; 5 International Clinical Research Center, St. Anne’s University Hospital Brno, Brno, Czech Republic.; 6 Department of Pharmaceutical Sciences, University of Antwerp, Antwerp, Belgium.; 7 Department of Immunology and Respiratory Research, Boehringer Ingelheim Pharmaceuticals Inc., Ridgefield, Connecticut, USA.; 8 Department of Abdominal Surgery, University Hospitals Leuven, Leuven, Belgium.; 9 International Clinical Research Center, Faculty of Medicine, Masaryk University, Brno, Czech Republic.; 10 Institute of Hematology and Blood Transfusion, Prague, Czech Republic.; 11 Department of Microbiology, Immunology and Transplantation, KU Leuven, Leuven, Belgium.; 12 Department of Biomedical Sciences, Humanitas University, Milan, Italy.; 13 Department of Gastroenterology-Hepatology, Leiden University Medical Center, Leiden, Netherlands.; 14 Department of Gastroenterology and Hepatology, University Hospitals Leuven, Leuven, Belgium.; 15 Laboratory of Pathology, University Hospitals Leuven, Leuven, Belgium.; 16 Laboratory of Translational Cell and Tissue Research, Department of Imaging and Pathology, KU Leuven, Leuven, Belgium.; 17 Leuven Institute for Single Cell Omics (LISCO), KU Leuven, Leuven, Belgium.

**Keywords:** Gastroenterology, Inflammation, Fibrosis, Inflammatory bowel disease, Monocytes

## Abstract

Crohn’s disease (CD) is marked by recurring intestinal inflammation and tissue injury, often resulting in fibrostenosis and bowel obstruction, necessitating surgical intervention with high recurrence rates. To elucidate the mechanisms underlying fibrostenosis in CD, we analyzed the transcriptome of cells isolated from the transmural ileum of patients with CD, including a trio of lesions from each patient: non-affected, inflamed, and stenotic ileum samples, and compared them with samples from patients without CD. Our computational analysis revealed that profibrotic signals from a subset of monocyte-derived cells expressing CD150 induced a disease-specific fibroblast population, resulting in chronic inflammation and tissue fibrosis. The transcription factor TWIST1 was identified as a key modulator of fibroblast activation and extracellular matrix (ECM) deposition. Genetic and pharmacological inhibition of TWIST1 prevents fibroblast activation, reducing ECM production and collagen deposition. Our findings suggest that the myeloid-stromal axis may offer a promising therapeutic target to prevent fibrostenosis in CD.

## Abstract 2

Crosstalk between profibrotic fibroblasts and inflammatory monocytes regulates fibrostenosis in Crohn’s disease.

## Graphical abstract

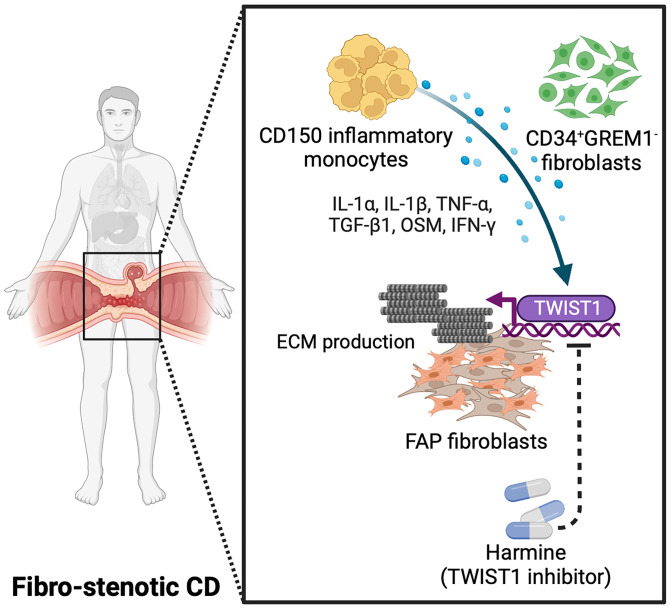

## Introduction

Crohn’s disease (CD) is a chronic inflammatory condition affecting the gastrointestinal tract, characterized by transmural inflammation that often leads to complications such as strictures, fistulas, and abscesses. The disease presents with considerable variability in its presentation, severity, and progression. Although patients with CD share common clinical characteristics, the natural disease course is rather heterogeneous; the disease can remain indolent or progress rapidly toward severe comorbidities (1–3). Strictures develop in approximately 30% to 40% of CD patients over time, leading to surgery in 70% of cases and recurrent postoperative complications in nearly 40% within a decade (4–6). Ileal stricturing results from excessive extracellular matrix (ECM) deposition and muscular hyperplasia due to repeated cycles of inflammation and tissue repair, leading to fibrosis and bowel obstruction, commonly referred to as fibrostenosis (7, 8).

Current CD therapies aim to suppress inflammation through the administration of corticosteroids, immunosuppressive agents, and/or biologicals such as anti–tumor necrosis factor (anti-TNF) antibodies, anti-integrins, anti–IL-12/23 agents, or JAK inhibitors (9). While these therapies lead to symptomatic disease remission in approximately 40% of patients, recurrent flares still cause cumulative tissue damage and remodeling of the gut wall, often leading to fibrostenosis (10). As no antifibrotic drugs are currently available for CD patients, the incidence of fibrostenosis, and consequently that of surgical resection, remain very high (11). Most of the existing research on mucosal inflammation and cellular heterogeneity in inflammatory bowel disease (IBD) has relied on biopsies procured through ileocolonoscopy, which do not include the deeper layers of the gut (12–17). Consequently, these studies have been limited by their sampling methodology, failing to address transmural inflammation and fully elucidate the underlying causes of bowel remodeling and obstruction in the ileum and colon of CD patients.

Our study used single-cell RNA sequencing (scRNA-Seq) to analyze full-thickness transmural terminal ileum samples from fibrostenotic CD patients undergoing ileocecal resection. We profiled 3 stages of lesions: proximal non-affected ileum, inflamed ileum with ulceration, and stenotic ileum, aiming to characterize disease progression. Our study extends the knowledge on cell heterogeneity in the transmural ileum of fibrostenotic CD patients and highlights complex intercellular interactions between immune cells and fibroblasts, driving the process of tissue remodeling and the establishment of fibrosis in CD. We found that during inflammation and stenosis, pathogenic CD150^+^ inflammatory monocytes promote tissue remodeling and fibrosis by inducing the differentiation of FAP^+^ fibroblasts, leading to excessive ECM deposition via the transcriptional regulator TWIST1. Overall, our findings suggest that targeting the myeloid-mesenchymal axis during inflammation could be an effective strategy to prevent fibrostenosis in patients with CD.

## Results

### Uncovering the heterogeneity of fibroblasts in fibrostenotic CD.

Even though CD is a transmural disease, the use of endoscopic sampling has been a persistent limitation in previous studies (15–18). Therefore, we aimed to investigate the cellular landscape and intercellular interactions in the transmural ileum of CD patients to identify potential therapeutic targets. First, we classified transmural biopsies from resected ileal tissue within each patient based on macroscopic features. Then, we determined inflammatory and fibrotic activity in the proximal healthy margin and inflamed and stenotic ileum of CD patients, and control non-CD healthy ileum from colorectal cancer (CRC) patients, using hematoxylin and eosin (H&E) and Masson’s trichrome staining (Supplemental Figure 1A; supplemental material available online with this article; https://doi.org/10.1172/JCI173835DS1). To evaluate our sample classification microscopically, we modified a histopathological scoring system based on previous studies to assess inflammation and fibrostenosis in CD ileum (Supplemental Methods) (19, 20). We observed moderate to severe degrees of inflammation and fibrosis, including fissuring ulceration and abscess in inflamed ileum and stenotic ileum (Supplemental Figure 1B, left and middle). Although similar fibrosis features were found in both inflamed and stenotic ileum, stenotic ileum had a significantly higher level of overall collagen deposition (Supplemental Figure 1A, shown in blue) compared with inflamed ileum (*P* < 0.05) (Supplemental Figure 1B, right). Altogether, these findings confirmed that our method of classification was reliable to study the progression of tissue through inflammation to fibrostenosis.

**Figure 1:**
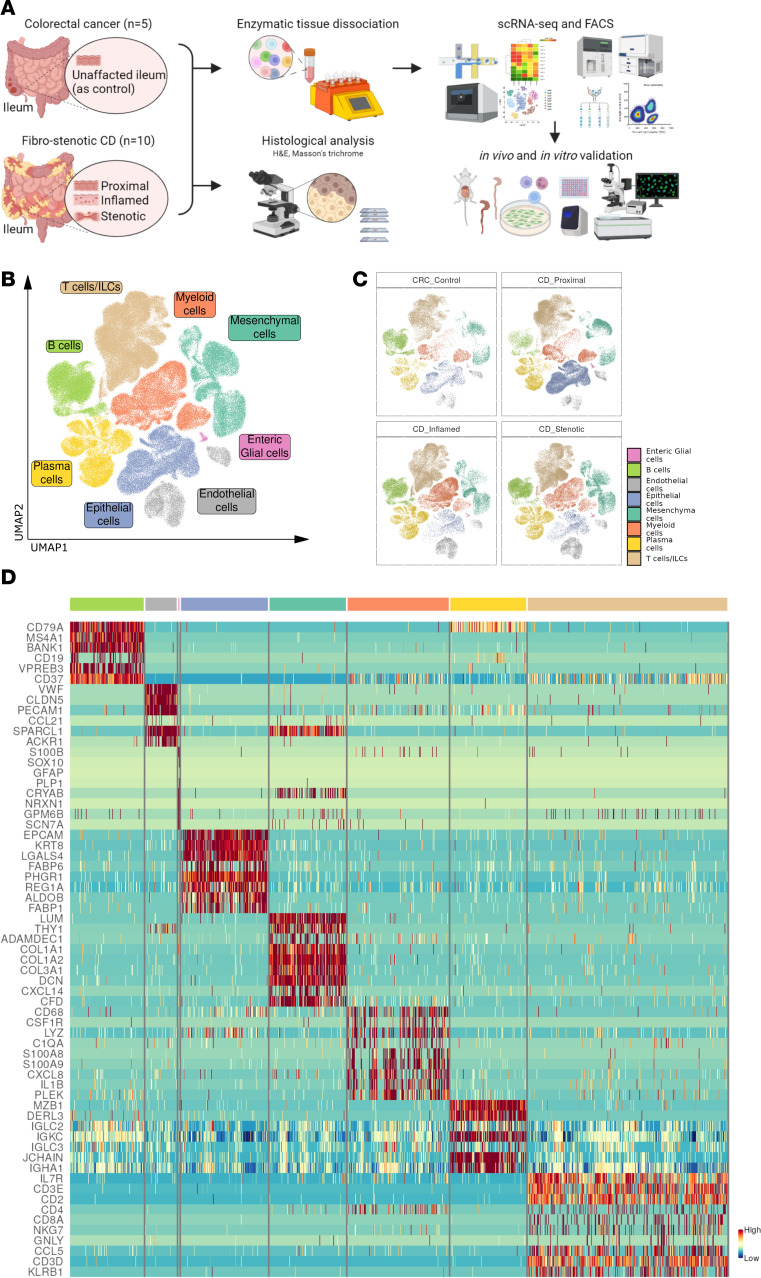
Single-cell profiling of fibrostenotic ileum from CD and control ileum from CRC. (**A**) Experimental workflow for scRNA-Seq of ileum using the 10x Genomics Chromium platform and further analyses and validations in this study. (**B**) Uniform manifold approximation and projection (UMAP) embedding showing ileal single-cell transcriptomes from 169,547 cells from 10 CD patients with a trio of lesions (proximal, inflamed, and stenotic) and 5 CRC control ilea, depicting cell compartments. ILCs, innate lymphoid cells. (**C**) UMAP in **B** split by disease segments. (**D**) Heatmap depicting relative expression of distinguishing marker genes in each cell compartment.

To uncover the interplay between immune and stromal cells during fibrosis in CD, we profiled the transcriptome of 169,205 cells from transmural terminal ileum of CD patients (*n* = 10, a trio of lesions from each; proximal as unaffected, inflamed, and stenotic ileum) and CRC patients (*n* = 5; control ileum) using scRNA-Seq (Figure 1A). A similar number of cells were profiled from each of the 3 CD lesions: proximal, 33.78%; inflamed, 32.48%; stenotic, 33.77% (Supplemental Figure 1, C–E). Unsupervised clustering followed by annotation of the integrated gene expression data identified several clusters that were classified into 8 compartments: T cells and innate lymphoid cells (*CD3D*, *CD3E*, *CD4*, *CD8A*, *NKG7*, *GNLY*, *KLRB1*), B cells (*CD19*, *CD79A*, *MS4A1*), plasma cells (*IGKC*, *MZB1*, *JCHAIN*), myeloid cells (*CD68*, *LYZ*), epithelial cells (*EPCAM*), endothelial cells (*VWF*, *PECAM1*), enteric glial cells (*PLP1*, *S100B*), and mesenchymal cells (*LUM*, *PDGFRA*, *DCN*) (Figure 1, B–D). To identify the major ECM-producing cell compartments, we compared expression of ECM core genes between each cell compartment. This comparison confirmed the mesenchymal compartment as the major source of ECM proteins during inflammation and stenosis (Supplemental Figure 1F). Thus, we proceeded with a deeper characterization of the mesenchymal compartment to address their heterogeneity in different disease states.

**Figure 2:**
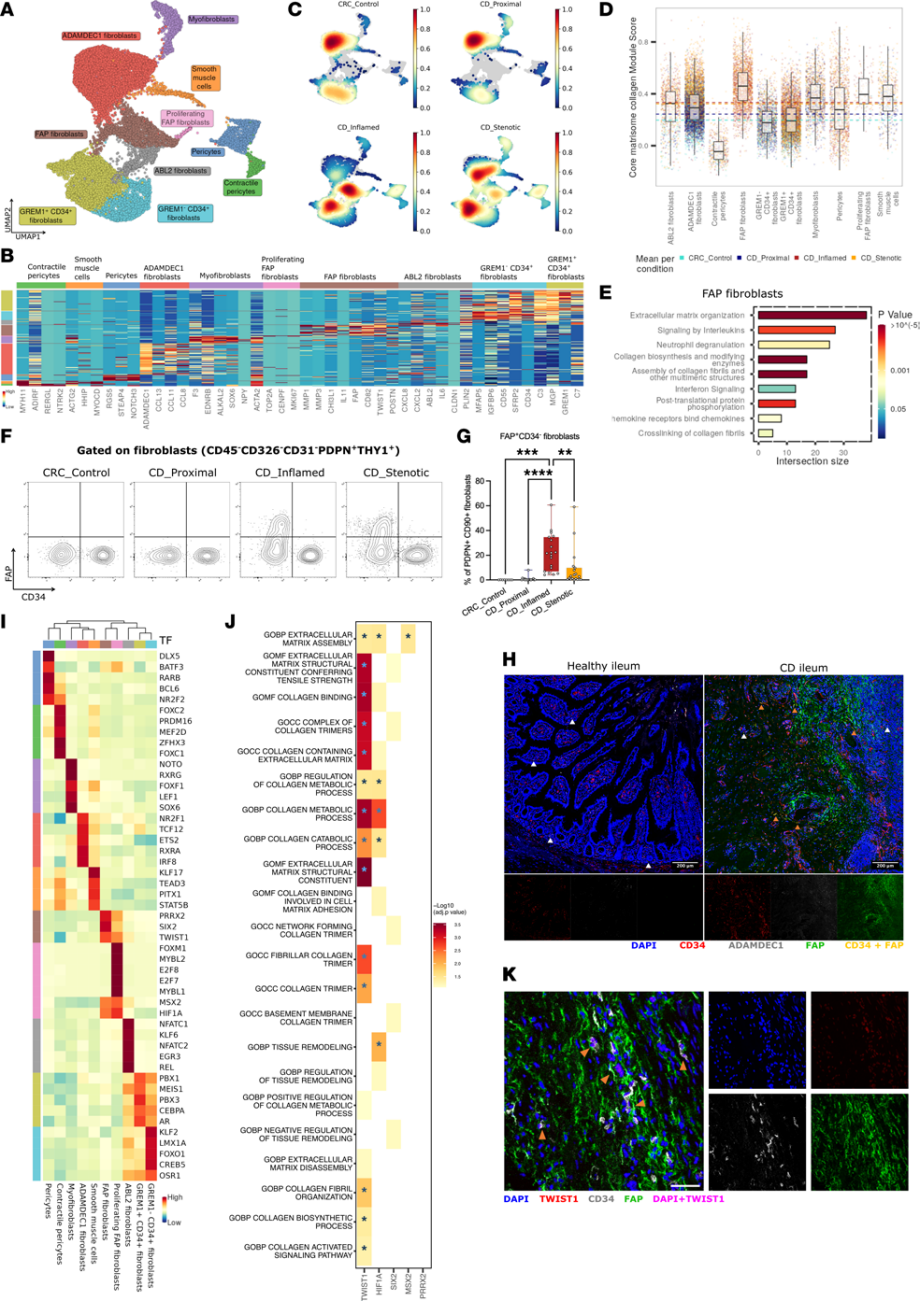
Heterogeneity of stromal cells in fibrostenotic CD. (**A**) UMAP representation of reclustered mesenchymal cells across different lesions of the terminal ileum. (**B**) Heatmap showing relative expression of top marker genes in each subset. (**C**) Cell subset composition across different lesions of the terminal ileum. (**D**) Bar plot showing gene set module score for core matrisome collagen genes in each stromal cell subset in different lesions. Horizontal lines indicate medians of respective lesions. (**E**) Enrichment analysis for Reactome biological pathways in FAP^+^ fibroblasts (log fold change > 0.5; FDR < 0.1). (**F** and **G**) Flow cytometry gating strategy for fibroblast subsets (**F**) and plot of FAP expression in pan-fibroblasts (7-AAD^–^CD45^–^CD31^–^CD326^–^PDPN^+^THY1^+^) (**G**) in different lesions of terminal ileum from 19 CD patients and 8 CRC control ilea. Data are shown as box-and-whisker plots. Statistically significant differences were determined using a 1-way ANOVA test corrected with Tukey’s multiple-comparison test (**P <0.01, ***P <0.005, ****P <0.001). (**H**) Immunofluorescence staining for PDPN, ADAMDEC1 (indicated by white arrowheads), CD34, and FAP expression in healthy ileum and CD diseased ileum. CD34 and FAP colocalization is indicated by orange arrowheads (scale bars: 200 μm). (**I**) Heatmap showing relative transcription factor activity in each stromal cell subset based on single-cell regulatory network inference and clustering (SCENIC) analysis. (**J**) Heatmap showing selected terms after functional enrichment analysis of top 5 regulons using GO terms and core ECM gene set from MatrisomeDB (*statistically significant terms after 1-sided Fisher’s exact test and multiple correction by Benjamini-Hochberg method). (**K**) Immunofluorescence staining for TWIST1 and CD34 expression in FAP^+^ fibroblasts in CD diseased ileum (indicated by arrowheads; scale bar: 50 μm).

Reclustering of the mesenchymal compartment identified 2 clusters of mural cells: pericytes (*RGS5*) and contractile pericytes (*ADIRF*); one cluster of smooth muscle cells (*MYOCD*, *MYH11*, *ACTG2*); and 7 clusters of fibroblasts: myofibroblasts (*SOX6*, *ACTA2*), ADAMDEC1^+^ fibroblasts (*ADAMDEC1*, *CCL11*), ABL2^+^ fibroblasts (*ABL2*, *PLIN2*, *CLDN1*), GREM1^–^CD34^+^ fibroblasts (*MFAP5*, *CD55*), GREM1^+^CD34^+^ fibroblasts (*GREM1*, *C3*, *C7*), FAP^+^ fibroblasts (*FAP*, *CD82*, *TWIST1*, *POSTN*), and a cluster of proliferating FAP^+^ fibroblasts (*MKI67*, *TOP2A*, *CENPF*, *FAP*, *CD82*, *TWIST1*, *POSTN*) (Figure 2, A and B). FAP^+^ fibroblasts and ABL2^+^ fibroblasts were found to be unique to inflamed and stenotic ileum compared with control and proximal ileum (Figure 2C and Supplemental Figure 2A). To identify the stromal cluster responsible for pathological ECM deposition, we developed a collagen module score using the core matrisome collagen gene signature from MatrisomeDB. This approach identified FAP^+^ fibroblasts as the primary ECM-producing cells among all mesenchymal subsets (Figure 2D). This was confirmed by Gene Ontology (GO) enrichment analysis, which showed that FAP^+^ fibroblasts were significantly enriched in GO processes associated with ECM organization (Figure 2E).

Next, to confirm the presence of FAP^+^ fibroblasts in inflamed and stenotic samples, we performed flow cytometry on transmural ileum of CD patients. After gating out leukocytes (CD45), endothelial cells (CD31), and epithelial cells (CD326), we used CD90 and podoplanin (PDPN) to identify fibroblasts (Figure 2F and Supplemental Figure 2B). Using this approach, we confirmed that FAP^+^ fibroblasts (FAP^+^CD34^–^) were unique to the inflamed and stenotic ileum and absent in the control and proximal ileum (Figure 2G and Supplemental Figure 2, C and D). In line with this, FAP enzymatic activity was significantly elevated in the inflamed and stenotic CD ileum compared with unaffected margins (Supplemental Figure 2E) (21). In addition, we investigated the spatial distribution of the fibroblast subsets using multiplexed immunofluorescence staining (PDPN, CD34, FAP, and ADAMDEC1). Importantly, we observed high expression of FAP in the submucosa and deeper layers of CD ileum where excess ECM deposition is observed. On the other hand, ADAMDEC1^+^ fibroblasts were predominantly present in the healthy mucosa (Figure 2H and Supplemental Figure 2F).

The FAP^+^ fibroblasts not only expressed ECM genes more highly but were also characterized by an activated phenotype with overexpression of CD90, PDPN, and FAP proteins, profibrotic autocrine loop molecules (*IL6*, *IL11*, *TGFB1*), neutrophil-chemoattracting chemokines (*CXCL1*, *CXCL5*, *CXCL6*), and monocyte-chemoattracting chemokines (*CCL2*, *CCL5*, *CCL7*) (Supplemental Figure 2G). Moreover, single-sample gene set enrichment analysis (ssGSEA) confirmed that FAP^+^ fibroblasts were enriched for processes such as inflammatory response and leukocyte chemotaxis (Supplemental Figure 2H) (22). Overall, these results suggested a key role for FAP^+^ fibroblasts in perpetual recruitment and potential activation of myeloid cells into the tissue during chronic inflammation.

Eventually, we compared our data with previously published IBD scRNA-Seq data sets of mucosal biopsies from CD and ulcerative colitis (UC) patients using Seurat integration and label transferring (Supplemental Figure 8A) (15–17). Cross-data-set cell type prediction score showed low to moderate similarity of FAP^+^ fibroblasts across these 3 data sets (Supplemental Figure 2I). To further validate the presence of FAP^+^ fibroblasts across both CD and UC in colon, we analyzed transmural samples from healthy CRC colon (unaffected), healthy CD colon (non-inflamed), inflamed/stenotic CD colon (granulating ulcer and thickened bowel wall), and UC colon (inflamed) using flow cytometry (Supplemental Figure 2J). Notably, a high proportion of FAP^+^ fibroblasts was predominantly observed in the inflamed/stenotic colons of patients with CD rather than in patients with inflamed colons due to UC (Supplemental Figure 2K). These findings imply that FAP^+^ fibroblasts represent a distinct pathogenic subset that emerges during chronic inflammation and fibrosis, characterized by excessive ECM deposition in CD.

### Transcriptional regulation and proinflammatory properties of FAP^+^ fibroblasts.

Given the unique involvement of FAP^+^ fibroblasts in ECM deposition, we set out to investigate the upstream transcriptional regulators driving their transcriptional pathological state. Using single-cell regulatory network inference and clustering (SCENIC) analysis, TWIST1, SIX2, PRRX2, MSX2, and HIF1α were identified as the top regulons (a network of a transcription factor and corresponding targets) active in FAP^+^ fibroblasts (Figure 2I) (23). When gene enrichment analysis of top regulons was performed, TWIST1 target genes stood out with significant enrichment of several GO terms associated with excessive ECM deposition such as collagen-containing ECM as well as the gene set containing the core constituents of ECM (Figure 2J). Using multiplex immunofluorescent staining (CD34, FAP, and TWIST1), we confirmed that in the inflamed ileum of CD patients, TWIST1 was expressed in the nuclei of FAP-expressing fibroblasts (Figure 2K).

Consistent with the established role of TWIST1 in epithelial-mesenchymal transition (EMT), ssGSEA demonstrated a significant enrichment of EMT gene signatures in FAP^+^ fibroblasts (Supplemental Figure 2L) (24, 25). In addition, our ssGSEA analysis revealed a significant enrichment of terms associated with cellular senescence, including KEGG’s cellular senescence and Reactome’s senescence-associated secretory phenotype in FAP^+^ fibroblasts (Supplemental Figure 2M), and enrichment of genes associated with the senescence-associated secretory phenotype (Supplemental Figure 2N) (26–28). Overall, these results suggest that TWIST1 drives a profibrotic phenotype in FAP^+^ fibroblasts and activates cellular senescence pathways.

### GREM1^–^CD34^+^ fibroblasts as potential precursors of FAP^+^ fibroblasts.

**Figure 3:**
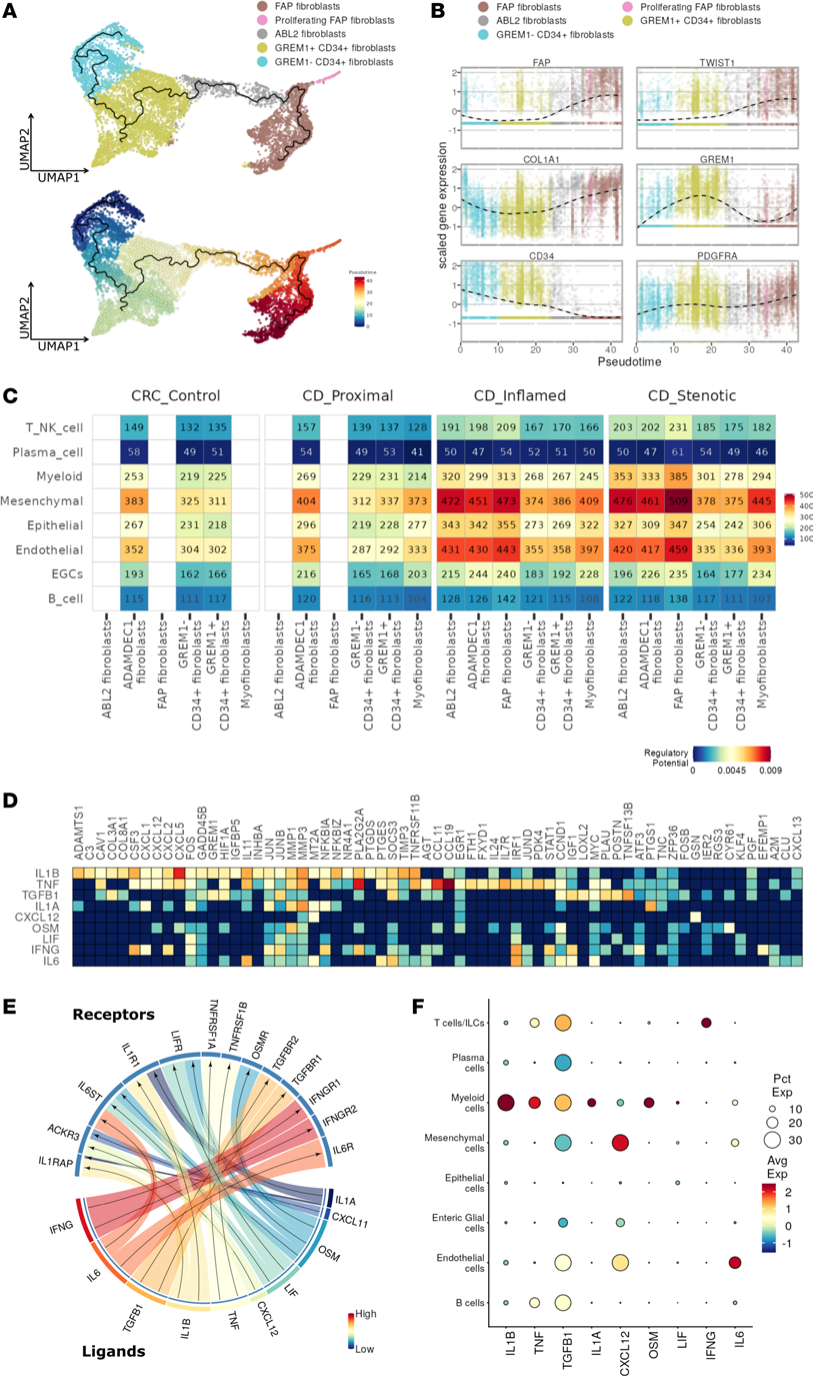
Trajectory analysis of fibroblast subset and stromal-immune interactions. (**A**) Pseudotime trajectory projected onto a UMAP of selected fibroblast subsets. (**B**) Normalized expression levels of selected markers visualized along the pseudotime. (**C**) Heatmap showing number of interactions (ligand-receptor pairs) between cell compartments and mesenchymal subsets. (**D**) Niche signaling driving FAP^+^ fibroblast differentiation, predicted by NicheNet; regulatory potential of each target gene in columns by ligands in rows. (**E**) Circos plot depicting links between predicted ligands by NicheNet and their receptors. (**F**) Dot plot showing expression of NicheNet-predicted ligands in all cell compartments.

To gain insight into how FAP^+^ fibroblasts appear during chronic inflammation, we exploited the gene expression data to construct a differentiation trajectory for FAP^+^ fibroblasts. After identifying 3 fibroblast clusters topologically connected to FAP^+^ fibroblasts using partition-based graph abstraction (PAGA), we applied Monocle 3 on these 4 clusters (Supplemental Figure 3A) (29, 30). Trajectory analysis revealed that GREM1^–^CD34^+^ fibroblasts differentiated into FAP^+^ fibroblasts during inflammation (Figure 3A). Along the differentiation trajectory toward FAP^+^ fibroblasts, fibroblasts exhibited a loss of CD34 expression and an acquisition of *TWIST1*, *FAP*, and *COL1A1* expression (Figure 3B and Supplemental Figure 3B). Multiplex immunofluorescent staining also showed the expression of CD34 in FAP^+^ fibroblasts together with TWIST1, indicating the differentiation status (Figure 2K). We hypothesized that the markedly distinct inflammatory microenvironment in CD may induce FAP^+^ fibroblast differentiation from GREM1^–^CD34^+^ fibroblasts. To identify the specific factors driving this differentiation, we used CellPhoneDB, a computational tool that estimates intercellular interactions between cell types and fibroblasts based on a curated database of ligand-receptor interactions (31). Using this approach, we observed an intense crosstalk within the mesenchymal compartment and between mesenchymal, endothelial, and myeloid cells. Notably, myeloid cells were responsible for most of the predicted interactions with the disease-associated mesenchymal cells in general and particularly with FAP^+^ fibroblasts as lesions progressed from inflammation to stenosis (Figure 3C and Supplemental Figure 3C).

### Stromal-immune cell interaction induces FAP^+^ fibroblast differentiation and activation.

To better understand the signaling cues involved in the transcriptional shift to FAP^+^ fibroblasts in CD ileum, we used a recently described computational tool called NicheNet, which enables the prioritization of ligands responsible for inducing alterations in a specific gene set (32). Based on the differentially regulated genes between GREM1^–^CD34^+^ fibroblasts and FAP^+^ fibroblasts, NicheNet predicted *IL1A*, *IL1B*, *IL6*, *CXCL12*, *IFNG*, *LIF*, *OSM*, *TNF*, and *TGFB1* as the top ligands associated with the transcriptional reprogramming of GREM1^–^CD34^+^ fibroblasts toward FAP^+^ fibroblasts (Figure 3, D and E). Next, we compared the gene expression levels of these ligands between cell compartments, and we found that the myeloid compartment exhibited the highest gene expression levels of the highest-ranked ligands, including *IL1A*, *IL1B*, *TNF*, *TGFB1*, and *OSM*. In contrast, *IFNG* expression was predominantly found in T cells/innate lymphoid cells, while *IL6* and *CXCL12* were mainly expressed by mesenchymal and endothelial cells (Figure 3F and Supplemental Figure 3C). The activation of these signaling pathways was also confirmed by ssGSEA analysis (Supplemental Figure 3D). Taken together, ligand-target analysis suggested myeloid cells as the major source of profibrotic ligands, leading to FAP^+^ fibroblast cell state differentiation and activation.

### scRNA-Seq analysis reveals presence of a profibrotic monocyte subset specific to inflammation and stenosis.

**Figure 4:**
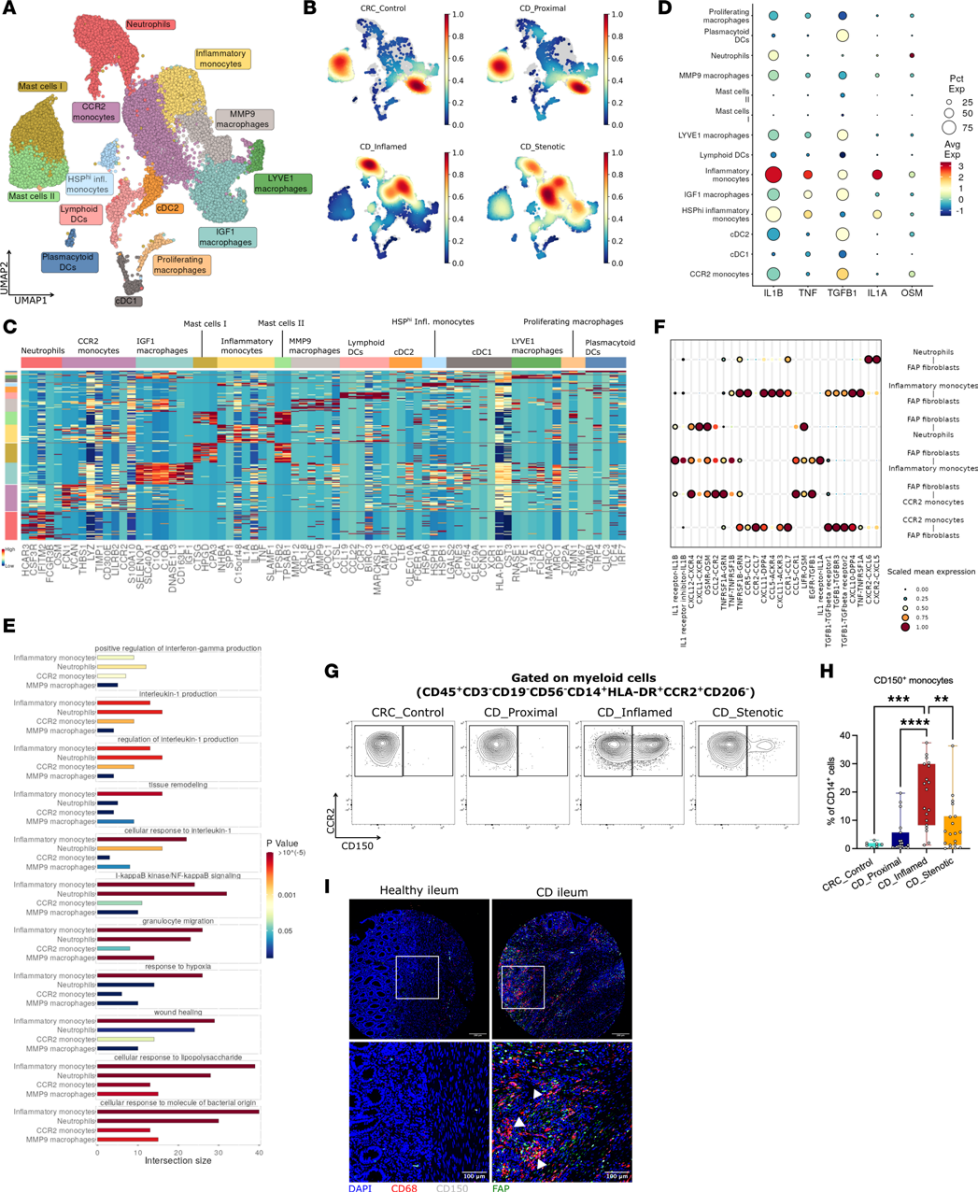
Heterogeneity of myeloid cells in fibrostenotic CD. (**A** and **B**) UMAP representation of reclustered myeloid cells (**A**) and cell subset composition (**B**) across different lesions of the terminal ileum. (**C**) Heatmap showing the expression of the top marker genes of each myeloid subset. (**D**) Dot plot showing NicheNet-predicted ligands expressed by myeloid cell subsets. (**E**) Selected GO terms significantly enriched in myeloid cell subsets. (**F**) CellPhoneDB dot plot showing ligand-receptor interactions between FAP^+^ fibroblasts and inflammatory monocytes or neutrophils. First and second interacting molecules correspond to first and second cell types on the *y* axis, respectively. Black circles indicate significant interactions. (**G** and **H**) Flow cytometry gating strategy for myeloid cell subpopulations (**G**) and plot of CD150 (*SLAMF1*) expression (**H**) in CD14^+^ myeloid cells (7-AAD^–^CD45^+^CD3^–^CD19^–^CD56^–^HLA-DR^+/–^) in different lesions of terminal ileum from 19 CD patients and 8 CRC control ilea. Data are shown as box-and-whisker plots. Statistically significant differences were determined using a 1-way ANOVA test corrected with Tukey’s multiple-comparison test (**P <0.01, ***P <0.005, ****P <0.001). (**I**) Immunofluorescence staining for CD68, CD150, and FAP expression in healthy ileum and CD diseased ileum. Original image composed of stitched ×25 images. Scale bars: 200 μm in top panels, 100 μm in bottom panels. White arrowheads indicate the spot of colocalization.

To identify the profibrotic myeloid cell subsets involved in driving the activation of FAP^+^ fibroblasts, we performed unsupervised reclustering of the myeloid compartment (*LYZ*, *CTSG*, *CD68*, *CSF1R*), and we identified a total of 14 clusters in the CD ileum and in the healthy CRC control ileum (Figure 4, A and B, and Supplemental Figure 4A). Two clusters of mast cells (*KIT*, *CTSG*) were identified in all segments, with minimal differences in gene expression observed between the 2 clusters (data not shown). Four distinct dendritic cell (DC) clusters were also identified: cDC1 (*CCND1*, *CLEC9A*); cDC2 (*LTB*); lymphoid DCs (*CCR7*, *LAMP3*), which were enriched in inflammation; and plasmacytoid DCs (pDCs), which initially clustered along with B cells (*GZMB*, *TCF4*, *IRF7*) (Figure 4C). The macrophage and monocyte clusters exhibited remarkable variations across the different tissue conditions. Two macrophage clusters, distinguished by *LYVE1* or *IGF1* expression, were mainly present in non-inflamed ileum as opposed to inflamed and stenotic ileum. These clusters also displayed other typical mature resident macrophage markers such as *C1QA* and *C1QB* but lacked *CCR2* expression. Three clusters, namely CCR2^+^ monocytes, MMP9^+^ macrophages, and neutrophils, were predominant in inflamed and stenotic ileum (Supplemental Figure 4A). We also observed an inflammatory monocyte cluster (“inflammatory monocytes”), which could be identified by the expression of *SLAMF1* (CD150) and common monocyte markers, including *CD300E*, *FCN1*, and *CCR2*. Most strikingly, this cell subset was exclusively present in inflamed and stenotic ileum (Figure 4B and Supplemental Figure 4A). To identify the subset(s) of myeloid cells inducing FAP^+^ fibroblast differentiation during chronic inflammation, we evaluated the inflammation-specific appearance and secretion of myeloid-derived profibrotic ligands. Most importantly, inflammatory monocytes had the highest expression of the profibrotic ligands (*IL1A*, *IL1B*, *TNF*, and *TGFB1*) predicted by the NicheNet analysis (Figure 4D).

### Inflammatory monocytes as a hyperinflammatory activation state of monocytes.

Given the potential role of inflammatory monocytes in fibrosis, we compared the transcriptional profile of the 4 myeloid clusters (neutrophils, CCR2^+^ monocytes, inflammatory monocytes, and MMP9^+^ macrophages) predominantly present in inflamed and stenotic ileum. The functional gene enrichment analysis using GO terms revealed a significant enrichment in inflammation- and fibrosis-associated GO terms such as (regulation of) interleukin-1 production, tissue remodeling, and wound healing, indicating their high inflammatory and profibrotic profile compared with the other 3 myeloid subsets (Figure 4E). Similarly, Reactome pathway analysis revealed enrichment of pathways associated with ECM organization, binding of chemokine receptors by chemokines, and IL-1 family signaling in inflammatory monocytes. This underscores the inflammatory and profibrotic characteristics of these cells (Supplemental Figure 4B). Furthermore, we quantified the intercellular interactions between inflammatory monocytes and other cell types using CellPhoneDB. Our analysis revealed that FAP^+^ fibroblasts exhibited a higher number of ligand-receptor interactions with inflammatory monocytes in comparison with other immune cell subsets (Figure 4F and Supplemental Figure 4C). Notably, this analysis indicated a potential role for FAP^+^ fibroblasts in the recruitment of monocytes via CCL2, CCL5, and CCL7 (Supplemental Figure 2G and Figure 4F).

Further, to distinguish the main transcription factors driving the profibrotic phenotype of inflammatory monocytes, we performed SCENIC analysis. ELK3, MSC, STAT4, HIF1α, and IRF7 were found to be among the top significantly enriched regulons in inflammatory monocytes (Supplemental Figure 4D). To understand the monocyte dynamics across disease states, we performed trajectory analysis of clusters connected with monocytes as identified by PAGA (data not shown). Nevertheless, we removed pDCs from the trajectory analysis, as they have been reported to be not of monocyte origin (33). Trajectory analysis showed 2 branches of monocyte differentiation through pseudotime (Supplemental Figure 4, E and F). One branch of CCR2^+^ monocytes gave rise to IGF1^+^ macrophages, with a subsidiary branch giving rise to cDC2. This branch was predominant in control and proximal ileum. The other branch depicted CCR2^+^ monocytes differentiating into inflammatory monocytes and further into MMP9^+^ macrophages and was predominant in inflamed and stenotic ileum (Figure 4B and Supplemental Figure 4, E and F).

### Inflammatory monocytes are predominantly present in inflamed and stenotic ileum.

To definitely confirm the association of the inflammatory monocytes with inflammation and stenosis in transmural CD ileum, we performed flow cytometry and immunofluorescence staining, using CD150 as a reliable marker to identify inflammatory monocytes within the myeloid compartment. In the diseased ileum, CD14^dim^ cells (as neutrophils), CD150^–^ monocytes (as CCR2 monocytes, CCR2^+^ and CD150^–^), and CD150^+^ monocytes (as inflammatory monocytes, CCR2^+^ and CD150^+^) were significantly increased, in comparison with the control and proximal ileum (*P* < 0.005) (Figure 4, G and H, and Supplemental Figure 4, G and H). Similarly, with multiplex immunofluorescent staining, we observed an increase of CD68^+^CD150^+^ cells (as inflammatory monocytes) in the deeper muscularis layer of diseased CD ileum, which colocalized with FAP expression (Figure 4I and Supplemental Figure 4I). We confirmed CD150 as a surface marker for inflammatory monocytes within the myeloid compartment, facilitating their isolation for in vitro experiments. Taken together, these findings indicate that inflammatory monocytes represent a pathogenic subset of myeloid cells secreting excess of inflammatory and profibrotic mediators and promoting differentiation of FAP^+^ fibroblasts and tissue remodeling.

To explore the potential of CD150 as a biomarker for CD in blood, we conducted flow cytometry analysis to assess the abundance of CD150^+^ monocytes in peripheral blood mononuclear cells (PBMCs). Notably, in patients with CD undergoing surgery for fibrostenosis, we found a significant increase in CD150^–^ circulating monocytes (Supplemental Figure 4M), but we did not observe an elevation in CD150^+^ monocyte levels in PBMCs.

### CD150^+^ inflammatory monocytes represent a specific feature of CD.

To contextualize our findings within the broader IBD research landscape, we reanalyzed our data and compared them with 3 IBD scRNA-Seq data sets derived from mucosal biopsies of both CD and UC patients using Seurat integration and label transferring (Supplemental Figure 8B) (15–17). Similar myeloid clusters were identified in all 3 IBD data sets with low similarity score in UC patients and moderate similarity score in CD samples (Supplemental Figure 4J). To further confirm the presence of inflammatory monocytes across UC and CD, we performed flow cytometry analysis by using transmural colon from CD and UC patients (Supplemental Figure 4K). Notably, the presence of CD150^+^ monocyte population was unique to CD in the colon and absent during UC (Supplemental Figure 4K). Together with our findings in CD ileum, these results suggest that CD150^+^ inflammatory monocytes are a unique subset with highly inflammatory and profibrotic properties, specific to CD patients in both inflamed ileum and colon.

### Highly multiplex spatial transcriptomics reveals colocalization of FAP^+^ fibroblasts and inflammatory monocytes in the inflamed and stenotic ileum.

**Figure 5:**
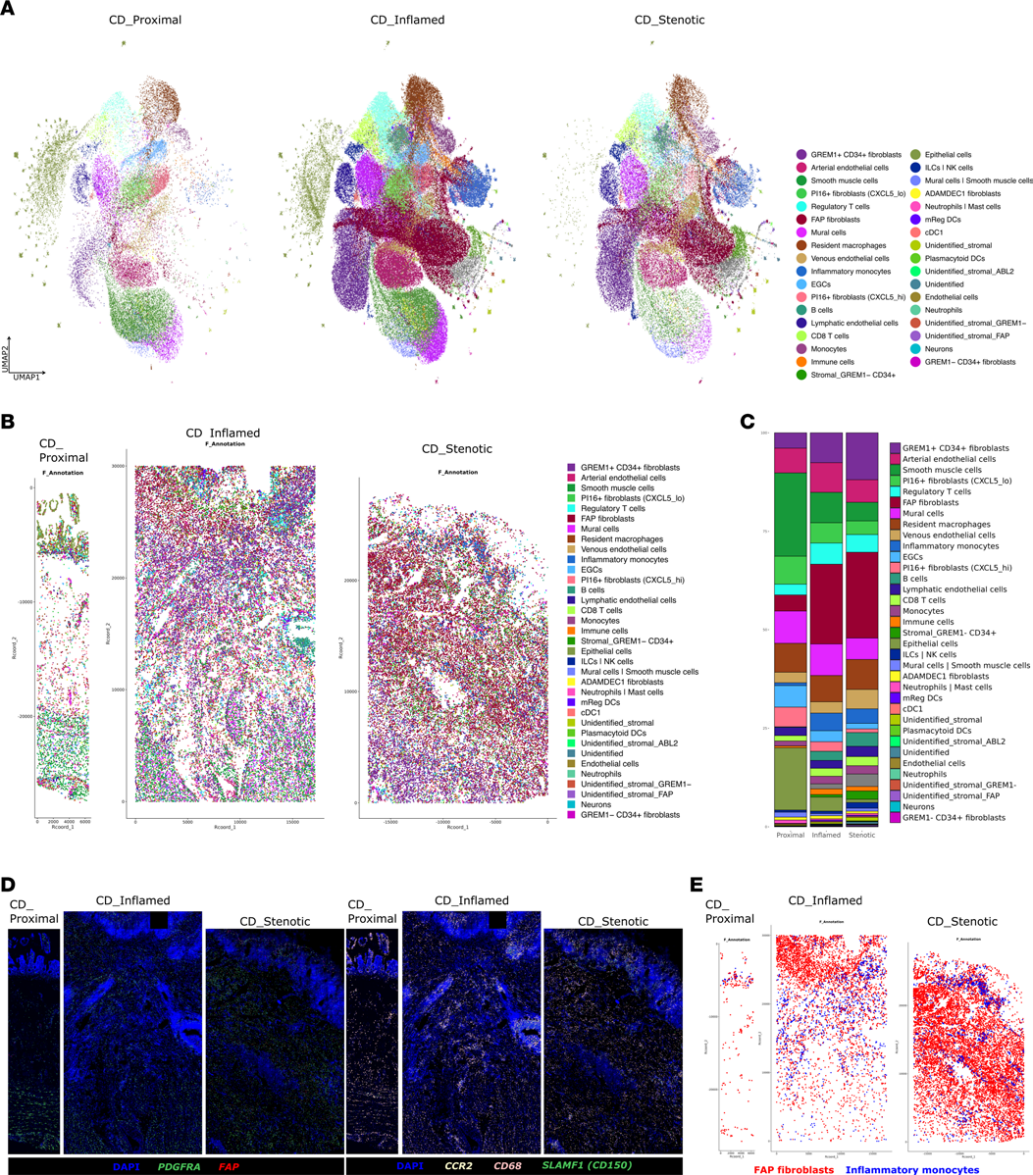
Spatial colocalization of FAP^+^ fibroblasts and inflammatory monocytes in inflamed and stenotic ileum of fibrostenotic CD patients. (**A**) UMAP representation of cell type across different lesions of the terminal ileum from 3 fibrostenotic CD patients. (**B**) Spatial map showing the location of cell types across different lesions of the terminal ileum. (**C**) Bar plot showing the proportion of cell types across different lesions of the terminal ileum. (**D**) Molecular Cartography of indicated genes in the full thickness of proximal and inflamed ileum and in the mucosa/submucosa layer of stenotic ileum. (**E**) Spatial map showing the colocalization of FAP^+^ fibroblasts and inflammatory monocytes in different lesions of terminal ileum.

In order to define the spatial distribution of cell clusters and delineate the fibroblast–myeloid cell niche in the CD-affected ileum, we conducted a multiplex spatial transcriptomic analysis on 99 genes (Supplemental Figure 5A) selected based on our scRNA-Seq data. We examined transmural terminal ileum samples from 3 CD patients, including proximal non-affected ileum, inflamed ileum with ulceration, and stenotic lesions. Upon cell segmentation analysis and unsupervised clustering, followed by annotation, we could observe the specific spatial locations of various cell types we identified in our scRNA-Seq. These included mesenchymal cells (*PDGFRA*, *PDGFRB*), myeloid cells (*CCR2*, *CD68*, *C1QA*), T cells (*CD3E*, *CD4*, *CD8A*), B cells (*CD19*, *MZB1*), epithelial cells (*ELF3*), endothelial cells (*ACKR1*, *CLDN5*, *PECAM1*, *VWF*), neurons (*UCHL1*), enteric glial cells (*PLP1*), and smooth muscle cells (*MYOCD*) (Figure 5A and Supplemental Figure 5A). The spatial map revealed the specific tissue locations of the disease-associated cell clusters (Figure 5B), confirming an increased proportion of FAP^+^ fibroblasts and inflammatory monocytes in the inflamed and stenotic ileum of CD patients (Figure 5C). Notably, these cells were absent in the healthy ileum of the same patients. Spatial localization of *FAP* and *PDGFRA* (FAP^+^ fibroblasts) and of *CCR2*, *CD68*, and *SLAMF1* (inflammatory monocytes) was primarily found in the submucosa of inflamed and stenotic ileum in CD patients (Figure 5D). The spatial map further underscored the close colocalization of FAP^+^ fibroblasts and inflammatory monocytes (Figure 5E). Collectively, these findings point toward intensive fibroblast–myeloid cell interactions during inflammation and fibrostenosis in the CD ileum.

### Inflammatory monocyte–derived profibrotic cues activate FAP^+^ fibroblasts via TWIST1.

**Figure 6:**
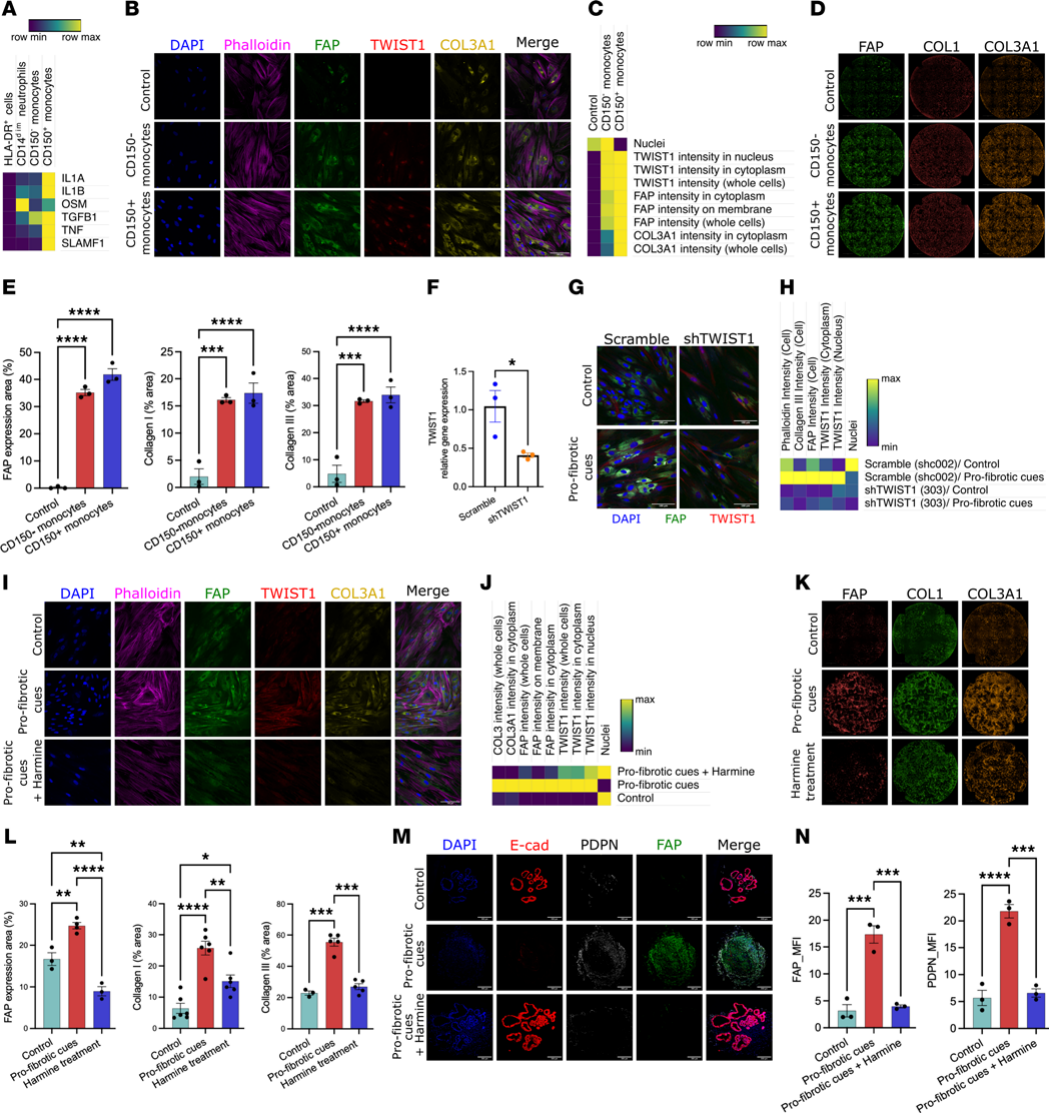
CD150^+^ monocyte-derived cytokines promote FAP^+^ fibroblast activation and ECM protein deposition under TWIST1 regulation. (**A**) Heatmap showing relative expression of NicheNet-predicted ligands expressed by FACS-sorted myeloid cell subsets (*n* = 4). (**B** and **C**) Immunofluorescence staining (**B**) and heatmap (**C**) showing relative expression of FAP, TWIST1, and type III collagen in monocyte-stimulated CCD-18Co fibroblasts (scale bar: 100 μm). (**D** and **E**) Immunofluorescence staining (original magnification, ×10) (**D**) and bar plot (**E**) showing relative expression of FAP and types I and III collagen in monocyte-stimulated CCD-18Co fibroblasts. Data are shown as bar plots with SEM. Statistically significant differences were determined using 1-way ANOVA test corrected with Tukey’s multiple-comparison test (***P <0.005, ****P <0.001) (scale bars: 1 mm). (**F**) Bar plot showing TWIST1 expression level after lentivirus transduction. Data are shown as bar plot with SEM. Statistically significant differences were determined using 2-tailed *t* test (*P <0.05). (**G** and **H**) Immunofluorescence staining (original magnification, ×25) (**G**) and heatmap (**H**) showing relative expression of FAP, TWIST1, and type III collagen in TWIST1-knockdown CCD-18Co fibroblasts stimulated by profibrotic cues (scale bars: 100 μm). (**I** and **J**) Immunofluorescence staining (**I**) and heatmap (**J**) showing relative expression of FAP, TWIST1, and type III collagen in CCD-18Co fibroblasts stimulated by profibrotic cues (scale bar: 100 μm). (**K** and **L**) Immunofluorescence staining (**K**) and bar plot (**L**) showing relative expression of FAP and types I and III collagen in profibrotic cues–stimulated CCD-18Co fibroblasts after TWIST1 inhibition. Data are shown as bar plot with SEM (scale bars: 1 mm). (**M** and **N**) Immunofluorescence staining (**M**) and quantitative analysis (MFI) (**N**) of FAP and PDPN in intestinal organoids derived from profibrotic cues–stimulated induced pluripotent stem cells, with or without harmine. Data are shown as bar plots with SEM. Statistically significant differences were determined using 1-way ANOVA test corrected with Tukey’s multiple-comparison test (*P <0.05, **P <0.01, ***P <0.005, ****P <0.001) (scale bars: 300 μm).

To validate the expression and functional relevance of profibrotic ligands in inflammatory monocytes as observed in our scRNA-Seq data, we quantified the gene expression of profibrotic ligands across different FACS-sorted myeloid subsets: HLA-DR^+^ cells, CD14^dim^ neutrophils (CD14^lo^, HLA-DR^–^), CD150^–^ monocytes, and CD150^+^ monocytes (Supplemental Figure 6A). In line with our scRNA-Seq data, inflammatory monocytes (CD150^+^ monocytes) showed the highest gene expression of *IL1A*, *IL1B*, *TNF*, *TGFB1*, and *SLAMF1* but not *OSM*, which was expressed mainly by the CD14^dim^ neutrophils as previously reported (Figure 6A) (18). Secretion of high levels of IL-1α, IL-1β, TNF-α, and TGF-β1 from the CD150^+^ monocytes was also confirmed at the protein level (Supplemental Figure 6B).

To confirm that inflammatory monocyte–derived ligands modulate fibroblast activation as seen in our scRNA-Seq data, we sorted CD14^dim^ neutrophils, CD150^–^ monocytes, and CD150^+^ monocytes from 5 CD patients undergoing surgery for ileal stenosis and collected cell supernatant after 16 hours of culture. Subsequently, the supernatants from FACS-sorted myeloid cells were used to stimulate CCD-18Co fibroblasts (CRL-1459, ATCC). In line with our computational prediction, the supernatants of inflammatory monocytes (CD150^+^ monocytes) isolated from inflamed ileum induced an activated fibroblast state with increased expression of TWIST1, FAP, and type III collagen compared with the supernatants of other FACS-sorted myeloid subsets (Figure 6, B and C, and Supplemental Figure 6C). To further confirm that ECM deposition induced by inflammatory monocyte–derived ligands modulates fibroblasts, we cocultured CCD-18Co fibroblasts and FACS-sorted myeloid cell supernatants with ascorbic acid for 3 weeks and performed immunofluorescent staining without permeabilization (Supplemental Figure 6D). Cell supernatants of inflammatory monocytes (CD150^+^ monocytes) promoted significantly higher FAP expression and higher extracellular deposition of type I and type III collagen by fibroblasts, in comparison with the control and CD14^dim^ neutrophils (Figure 6, D and E, and Supplemental Figure 6E).

Next, to assess the potency of different cytokine combinations in inducing fibroblast activation, we monitored the response of primary ileal fibroblasts isolated from transmural ileum, sourced from healthy regions of CRC patients, to different cytokine combinations using high-content imaging. The combination of IL-1α, IL-1β, TNF-α, TGF-β1, OSM, and IFN-γ, henceforth referred to as “profibrotic cues,” resulted in the highest expression of TWIST1, FAP, and type III collagen among different cytokine combinations after 48 hours of stimulation (Supplemental Figure 6F). Computational analysis had indicated a specific transcriptional regulatory role for TWIST1 in the excess ECM deposition exhibited by FAP^+^ fibroblasts. Consequently, we explored whether TWIST1 knockdown, achieved through lentivirus transduction, could influence fibroblast activation induced by profibrotic cues. Notably, we found that knockdown of TWIST1 expression significantly mitigated fibroblast activation and reduced collagen production in CCD-18Co fibroblasts (Figure 6, F–H).

To further validate whether pharmacological inhibition of TWIST1 could affect the profibrotic nature of activated fibroblasts, we used harmine, a recently identified TWIST1 inhibitor (34, 35). Following treatment with harmine, primary ileal fibroblasts stimulated by profibrotic cues exhibited a notable decrease in the expression levels of FAP, TWIST1, and type III collagen compared with those treated with the vehicle (Figure 6, I and J). Notably, harmine treatment also significantly reduced ECM deposition in ileal fibroblasts stimulated by profibrotic cues, with a marked decrease in the extracellular deposition of fibronectin and types I, III, and IV collagen, compared with vehicle-treated cells (Figure 6, K and L, and Supplemental Figure 6, H and I). Furthermore, using a 3D in vitro model based on intestinal organoids (IOs) that were derived from human induced pluripotent stem cells and comprised both epithelial and stromal cells, we demonstrated that profibrotic cues led to a significant increase in the expression of FAP and PDPN, which was substantially reduced in the presence of harmine (Figure 6, M and N). Altogether, our results provide solid evidence that inflammatory monocyte–derived profibrotic cues modulate FAP^+^ fibroblast activation and ECM secretion in a TWIST1-dependent manner.

### Genetic deletion or pharmacological inhibition of TWIST1 attenuates intestinal fibrosis induced by chronic colitis.

To verify the role of TWIST1 as a major driver of fibroblast activation and intestinal fibrosis in vivo, we generated fibroblast-specific Twist1-deficient mice (*Twist1*^Δ*/Δ*Col1a2^) by breeding Twist1-floxed mice (*Twist1^fl/fl^*) with *Col1a2*-CreER mice. Then we subjected *Twist1*^Δ*/Δ*Col1a2^ mice and their littermates to chronic dextran sulfate sodium (DSS) colitis upon tamoxifen administration. Notably, *Twist1*^Δ*/Δ*Col1a2^ mice showed a significant improvement in the overall disease activity index when compared with their littermates (Supplemental Figure 7, A–D). Additionally, *Twist1*^Δ*/Δ*Col1a2^ mice exhibited diminished collagen accumulation in the colon and reduced colon tissue size compared with their littermates (Supplemental Figure 7, E–G). In line with this, *Twist1*^Δ*/Δ*Col1a2^ mice after DSS-induced colitis also revealed significantly lower tissue damage as shown by the Mouse Colitis Histology Index (CHI) (Supplemental Figure 7, H–L). TWIST1 deletion in vivo was also associated with a reduction in FAP expression in fibroblasts isolated from the colon of *Twist1*^Δ*/Δ*Col1a2^ mice compared with their littermates after DSS-induced colitis (Supplemental Figure 7, M and N). In line with this, Cre recombinase deletion of TWIST1 in primary mouse colonic fibroblasts isolated from *Twist1^fl/fl^* mice resulted in lower induction of FAP and type III collagen expression in response to profibrotic cues (Supplemental Figure 7, O and P).

Finally, pharmacological blockade of TWIST1 with harmine led to diminished collagen deposition and reduced TWIST1 and FAP expression in the colon during chronic DSS colitis (*P* < 0.05), with no significant effect on the CHI (Supplemental Figure 7, Q–T) or colitis severity (Supplemental Figure 7, U–X). Our results confirmed that both genetic and pharmacological inhibition of TWIST1 mitigates gut fibrosis in chronic intestinal inflammation.

## Discussion

Lack of transmural sampling has been a long-standing limitation in CD studies even though the disease occurs in all layers of the gut wall (36). So far, scRNA-Seq studies on CD have mostly focused on characterizing mucosal inflammation using endoscopic biopsies and hence have not appreciated the transmural heterogeneity in CD ileum (15–17). In our study, we used scRNA-Seq to analyze transmural ileal biopsies from each fibrostenotic CD patient, covering a trio of lesions including proximal non-affected ileum, inflamed ileum with ulceration, and stenotic ileum. This approach allowed us to fully characterize the different stages of disease progression up to fibrostenosis within the deeper layers of the gut, where significant tissue remodeling is commonly observed in affected patients. Our data revealed a previously unknown heterogeneity in the transmural CD ileum, with the fibroblast and myeloid compartments showing remarkable differences across lesions. Deeper analysis of the mesenchymal compartment revealed FAP^+^ fibroblasts as the key pathogenic cell subset uniquely present in inflamed and stenotic CD ileum, responsible for excessive deposition of ECM. Notably, FAP^+^ fibroblasts were spatially enriched in the deeper submucosa and muscularis layers of fibrostenotic CD ileum in close proximity with inflammatory monocytes expressing *SLAMF1* (CD150). Additionally, FAP^+^ fibroblasts exhibited elevated expression of collagen and ECM genes, along with an activated phenotype characterized by overexpression of profibrotic autocrine loop molecules, such as *IL11* and *IL6*, and chemokines for neutrophils (*CXCL1*, *CXCL5*, *CXCL6*) and monocytes (*CCL2*, *CCL5*, *CCL7*), suggesting a critical role in perpetuating a feed-forward loop that sustains chronic inflammation within the tissue. Trajectory analysis indicates that FAP^+^ fibroblasts differentiate from homeostatic CD34^+^ GREM1^–^ fibroblasts during inflammation, which aligns with prior studies demonstrating that CD34 mesenchymal cells are essential for maintaining intestinal homeostasis and differentiate into myofibroblasts via the TGF-β/Smad2 signaling pathway in response to inflammation (37). Similar observations have been made in the heart, where depletion of CD34^+^ cells led to reduced myocardial fibrosis and improved cardiac function (38).

Transcriptional regulatory network analysis revealed TWIST1 as the main transcriptional regulator driving the excess ECM gene expression by FAP^+^ fibroblasts. TWIST1 has been primarily investigated in cancer for its role in EMT (24, 25). Concordantly, our gene enrichment analysis indicated an activation of ECM-related pathways in FAP^+^ fibroblasts. In line with our findings, TWIST1 has also been implicated in other fibrotic diseases, such as pulmonary fibrosis and renal fibrosis (39). Moreover, TWIST1 has demonstrated profibrotic properties in human fibroblasts by enhancing matrix stiffness (40). In our study, both pharmacological inhibition using harmine and genetic deletion of TWIST1 reduced fibroblast activation and ECM protein production in activated human and mouse intestinal fibroblasts (34, 35). Similar results were observed in a murine model of chemically induced chronic colitis, where TWIST1 deletion in fibroblasts (*Twist1*^Δ*/Δ*Col1a2^ mice) as well as treatment with harmine attenuated ECM deposition in the gut. The antifibrotic effects of TWIST1 deletion are in line with recent literature reporting reduced ECM accumulation in bleomycin-induced dermal fibrosis and skin wound healing (41, 42). Currently, several phase I studies are assessing the toxicity of harmine in patients (NCT05526430, NCT05780216, NCT05829603, and NCT04716335), and if successful, inhibition of TWIST1 via harmine may represent a future antifibrotic treatment, particularly for preventing intestinal fibrosis.

Overall, our findings are consistent with the evolving paradigm highlighting the crucial role of stromal cells, including FAP^+^ fibroblasts, in chronic inflammation and cancers across various organs such as the liver, lung, heart, and joints (43–48). Consistently, depletion of FAP^+^ fibroblasts using engineered FAP chimeric antigen receptor T cells resulted in reduced tissue damage and ECM deposition in experimental models of angiotensin II/phenylephrine–induced cardiac damage and bleomycin-induced lung fibrosis as well as reduced leukocyte infiltration and disease severity in a murine model of arthritis (49–51). Further, we looked into the environmental cues driving the differentiation of FAP^+^ fibroblasts and identified an extensive crosstalk between FAP^+^ fibroblasts and myeloid cells, in which myeloid-derived cytokines, including IL-1β, IL-1α, OSM, and TNF-α, promoted the differentiation of FAP^+^ fibroblasts (52). The plasticity of monocytes in response to the altered tissue microenvironment, and their capacity to direct stromal cells toward either a regulated wound healing process or dysregulated tissue remodeling and fibrosis have been widely described (53). In our study, inflammatory myeloid cells, differentiated from monocytes during inflammation, presented with a hyperinflammatory signature with high expression of *IL1A*, *IL1B*, and *TNF*. Reclustering of the myeloid cells revealed that proinflammatory ligands were secreted by a specific subset of monocytes identified by the unique expression of CD150, which colocalized with FAP^+^ fibroblasts in the deeper submucosa and muscularis layer in inflamed and stenotic CD lesions (54–56). Our observations are in line with a recent investigation demonstrating that, when stimulated with lipopolysaccharide, blood-derived monocytes from CD patients resistant to anti-TNF therapy display a hyperinflammatory phenotype, characterized by increased release of TNF-α, IL-23, and IL-1β (57). Taken together, these findings suggest a potential association between a dysregulated immune response in monocytes, activation of stromal cells, and resistance to anti-TNF therapy in certain CD patients with a high risk of developing fibrostenosis.

To contextualize our findings within the broader understanding of cellular heterogeneity in IBD, we compared our inflammatory monocyte and FAP^+^ fibroblast transcriptional signatures with those from recently published scRNA-Seq data sets (15–17). Despite including patients with B2-type stricturing CD, the signature scores for FAP^+^ fibroblasts and inflammatory monocytes in ileum samples from Kong et al.’s data were consistently low (16). In contrast, from the data of Martin et al., whose study used surgical samples from CD patients (15), we have identified high gene signature scores consistent with our FAP^+^ fibroblast and CD150^+^ monocyte subsets in the ileal lamina propria of a distinct subset of patients resistant to anti-TNF therapy. This discrepancy may be attributed to Kong et al.’s sampling strategy, which lacked transmural sampling (16). Thus, we postulate that the key difference between fibrostenosis and chronic inflammation in patients may lie in the localization of FAP^+^ fibroblasts in the deeper layers. In line with this, our pathological FAP^+^ stromal cell state resembled the ECM-high fibroblasts identified by Mukherjee et al. in full-thickness ileal samples as a major driver of stricture formation in CD patients (58). Eventually, and in line with data published by Smillie et al., we could not consistently identify activated FAP^+^ fibroblasts and CD150^+^ monocytes in transmural colonic samples from UC patients, suggesting that FAP^+^ fibroblasts and CD150^+^ inflammatory monocytes may predominantly represent a feature of CD (17).

Overall, our study extends our knowledge on cellular heterogeneity in the transmural ileum of fibrostenotic CD patients, highlighting key interactions between immune cells and fibroblasts. We discovered that inflammatory monocytes drive tissue remodeling and fibrosis by promoting via TWIST1 a profibrotic fibroblast state during inflammation and stenosis. Ultimately, our research has revealed multiple potential therapeutic targets, offering promise for developing more effective treatments for fibrostenotic CD.

## Methods

### Sex as a biological variable.

Sex was not considered as a biological variable; therefore, human and mouse studies included both sexes.

### Human specimens.

The resected terminal ileum was collected immediately after surgery from patients with Crohn’s disease (CD) or colorectal cancer (CRC) under the supervision of a specialized IBD pathologist. The healthy ileum was macroscopically classified as proximal tissue, while the ulcerative ileum exhibiting a non-thickened bowel wall was categorized as inflamed tissue. Conversely, the non-ulcerative ileum displaying a thickened bowel wall at the location of a narrowed lumen was designated as stenotic tissue.

### Histological slides of the terminal ileum.

Transmural biopsies from the terminal ileum were fixed in 4% formaldehyde and embedded in paraffin, and 5-μm-thick sections were cut for histological analysis. Hematoxylin and eosin (H&E) and Masson’s trichrome staining were performed in the Department of Imaging and Pathology at Universitair Ziekenhuis Leuven (UZ Leuven). The pathological score system was modified from Gordon et al., and examination was performed by a specialized IBD pathologist (19, 20). To quantify the relative histological area of collagen on Masson’s trichrome–stained slides, an average of 10 images in each sample were taken and quantified with ImageJ (NIH). The slides were imaged on a Marzhauser Slide Express 2 (Nikon) at the Vlaams Instituut voor Biotechnologie (VIB) Leuven.

### Single-cell isolation from the terminal ileum.

Single-cell suspensions were prepared from the transmural terminal ileum. Briefly, healthy (as the proximal tissue), granulating ulcerative (as the inflamed tissue), and thickened (as the stenotic tissue) biopsies of CD ileum and healthy (as the control group) ileum of CRC were treated with 1 mM DTT and 1 mM EDTA in 1× Hanks balanced salt solution (HBSS), followed by 1 mM EDTA in HBSS, respectively, at 37°C for 30 minutes. Then the tissue was minced and digested with 5.4 U/mL collagenase D (Roche Applied Science), 100 U/mL DNase I (Sigma-Aldrich), and 39.6 U/mL dispase II (Gibco) in a sterile gentleMACS C tube for 20 minutes at 37°C in a rotating incubator at 250–300 rpm followed by dissociation with the gentleMACS Dissociator (program human_tumor_02.01). After being treated with Red Blood Cell Lysis Buffer (11814389001, Roche), single-cell suspensions were used for scRNA-Seq, cell culture, and flow cytometry.

### Single-cell RNA sequencing and data analysis.

Cell suspensions were processed with a 10x Genomics 3′ v3 GEM kit and loaded on a 10x Genomics Chromium controller to create single-cell Gel Beads-in-emulsion (GEM). A cDNA library was created using a 10x Genomics 3′ v3 library kit and was then sequenced on a NovaSeq 6000 system (Illumina). Pre-processing of the samples including alignment and counting was performed using Cell Ranger Software from 10x Genomics.

Doublet score was calculated using 3 methods (scDblfinder, scrublet, and DoubletFinder) on each sample separately, and corresponding doublet scores were added to the metadata (59–61). Using the DropletQC package, a quality control (QC) metric indicating the fraction of reads exclusive to nuclear reads was also calculated (62). Thus, our QC metrics included fraction of nuclear reads, doublet scores, and unique molecular identifier (UMI).

Data were analyzed using Seurat v3 SCTransform-Integration workflow with each patient as a batch. Only cells with more than 199 unique genes and less than 30% mitochondrial genes were included in the analysis. Next, SCTransform function from Seurat was used to scale, normalize, and transform each sample (10x Genomics) with method set as “glmgGamPoi” and percentage of mitochondrial genes as the variable to regress (63–65). Next, 3,000 features were selected for integration, and a reference-based integration with 10 of the 35 samples as reference samples was performed. After principal component analysis (PCA), 80 principal components were used to find shared nearest neighbors and compute uniform manifold approximation and projection (UMAP). Next, the shared nearest neighbor graph was used to cluster the cells at a resolution of 2.

After clustering, 2 clusters (which clustered in the middle of the UMAP) with low UMI that did not express distinguishable markers for any cell type were removed. Some clusters, such as neutrophils, had a lower number of genes expressed but showed distinct neutrophil markers. A cluster with a high number of doublets as indicated by the doublet scores was also removed. Differential gene expression between clusters was performed using Wilcoxon’s test implemented in Seurat using FindAllMarkers or FindMarkers function.

### Annotation and subsetting of the data.

Small intestine data model pretrained on the human ileal single-cell data of the Human Gut Atlas was downloaded from the CellTypist website and was used to annotate the clusters with CellTypist (66, 67). The 73 clusters were then classified into 8 different compartments: mesenchymal, myeloid, T/NK cells, B cells, plasma cells, epithelial cells, endothelial cells, and enteric glial cells (EGCs), based on the CellTypist annotation. The annotation was additionally manually curated using canonical markers as shown in Figure 1D. Each compartment except EGCs was then separately reclustered to reveal detailed heterogeneity. First reclustering within each compartment at high resolution revealed low-quality cells and doublets, which were filtered out, and only high-quality cells were retained for the second round of reclustering.

Overall, an effort was made to stay consistent in annotation with earlier publication in the single-cell Human Gut Atlas (67). For the mesenchymal cells and myeloid cells, CellTypist annotation based on the Human Gut Atlas was used wherever possible. For T cells, B cells, epithelial cells, and plasma cells, manual annotation was performed after curation of CellTypist annotations based on 2 publicly available annotated scRNA-Seq data sets of similar tissue. After reclustering followed by filtering and annotation of subclusters in each compartment, all retained cells were used to compute the UMAP of all cells as in Figure 1B. The fine annotations of subclusters obtained from reclustering of each compartment were carried over to the metadata of all cells for downstream analyses such as CellPhoneDB.

### Gene regulatory network analysis.

Gene regulatory network analysis was performed using the Python implementation of single-cell regulatory network inference and clustering (SCENIC) (23). Specifically, GRNBoost was used to construct a gene regulatory network from log-normalized counts. Identified networks were then pruned using DNA motif analysis to remove indirect targets or associations, and enrichment of each regulon in single cells was quantified using the AUCell algorithm included in SCENIC. Further, Wilcoxon’s rank sum test as implemented in the Seurat R package was used to identify the top significant transcription factors in each cluster. The analysis was performed using pySCENIC (0.11.1), the Python implementation of SCENIC (68).

### Trajectory analysis.

PAGA was used to estimate the connectivity between the Seurat clusters (29). Four clusters including FAP^+^ fibroblasts identified by PAGA as connected were then used with Monocle 3 to learn the differentiation trajectory (30). Healthy tissue-specific GREM1^–^CD34^+^ fibroblasts were annotated as the root of the trajectory before ordering of cells along the pseudotime. Similarly, for myeloid cell trajectory, PAGA was used first to identify connected clusters. Connected clusters except pDCs were then used with Monocle 3 to construct the trajectory. Cells were ordered with CCR2^+^ monocytes at the beginning of pseudotime.

### Intercellular interaction and signaling analysis.

Overall ligand-receptor interactions among cell compartments were analyzed using CellPhoneDB (31). CellPhoneDB analysis was separately performed for each of the 4 tissue segments on all cells using subcluster annotations. Specific ligands involved in altering gene expression leading to differentiation of FAP^+^ fibroblasts were predicted using the NicheNet R package with the Kyoto Encyclopedia of Genes and Genomes (KEGG) database used for ligand-receptor pairs (26, 32). Genes differentially expressed between ABL2^+^ fibroblasts, GREM1^–^CD34^+^ fibroblasts, GREM1^+^CD34^+^ fibroblasts, and FAP^+^ fibroblasts were considered as the gene set of interest. Visualizations were prepared using the ggplot2 R package except for heatmap visualizations, which were prepared using the heatmap R package.

### In silico gene functional analysis.

Functional analysis of upregulated genes in specific clusters or a regulon was done using an enricher function from the ClusterProfiler package (69, 70). Also, for single-cell data of myeloid and mesenchymal compartments, single-sample gene set enrichment analysis (ssGSEA) as implemented in the scGSVA package was used with a combined database of KEGG, Reactome, Gene Ontology (GO), HALLMARK, BIOCARTA, and Human Phenotype (26, 28, 71–73). Wilcoxon’s test was used to estimate the statistical significance of the term enrichment in the clusters. For specific custom gene set of core matrisome collagens or core ECM genes, gene sets were downloaded from the MatrisomeDB database, and the AddModuleScore function from Seurat package was used to create a module score at the single-cell level (74).

### Flow cytometry, sorting, and analysis.

To validate cell proportion of scRNA-Seq in fibroblast subsets, cell suspensions were labeled with CD45-PE (1:300; clone 2D1, BioLegend), CD326-PE (1:300; clone 9C4, BioLegend), CD31-PE (1:300; clone WM59, BioLegend), CD90-BV421 (1:400; clone 5E10, BioLegend), podoplanin (PDPN)–APC (1:300; clone NC-08, BioLegend), CD34-FITC (1:200; clone 4H11, eBioscience), and FAP–Alexa Fluor 750 (1:300; clone 427819, R&D Systems). 7-Aminoactinomycin D (7-AAD) (1:100; 559925, BD Biosciences) was used to determine cell viability. The combination of CD45, CD31, and CD326 was used as lineage to eliminate immune cells, endothelial cells, and epithelial cells.

CD45-FITC (1:400; clone HI30, BioLegend), CD3–APC/Cy7 (1:300; clone UCHT1, BioLegend), CD56–APC/Cy7 (1:300; clone HCD56, BioLegend), CD19–APC/Cy7 (1:300; clone HIB19, BioLegend), HLA-DR–APC (1:300; clone L243, BioLegend), CD150 (SLAMF-1)–BV421 (1:100; clone A12, BD Horizon), CD14–PE/Cy7 (1:400; clone 63D3, BioLegend), CCR2-PE (1:400; clone LS132.1D9, BD Pharmingen), and CD206–PE/CF594 (1:300; clone 19.2, BD Horizon) were used to identify different subsets of myeloid cells. The combination of CD3, CD19, and CD56 was used as lineage to eliminate T cells, B cells, and NK cells. Flow cytometry and sorting were performed on the MA900 multi-application cell sorter (Sony) with 100 μm sorting chips.

Sorted HLA-DR^+^ cells, CD14^dim^ cells, CD150^–^CCR2^+^ cells, and CD150^+^CCR2^+^ cells (10,000 cells per well) were seeded in a 96-well clear round-bottom plate (3788, Corning) with 100 μL of RPMI 1640 medium and supplied with 5% FBS (BWSTS181H, VWR), 1% HEPES (15630056, Gibco), 1% l-glutamine (A2916801, Gibco), 1% sodium pyruvate (11360070, Gibco), and 1% antibiotic/antimycotic solution (A5955, Sigma-Aldrich) for 16 hours. The cell supernatants were collected and used to stimulate fibroblasts.

### Spatial transcriptomics.

Human ileum was embedded in Tissue-Tek O.C.T. Compound (Sakura) and snap-frozen in isopentane (Sigma-Aldrich) chilled by liquid nitrogen. Embedded tissues were stored at –80°C. Human ileum was sectioned and placed within capture areas on Resolve BioScience slides (8 × 8 mm). The samples were analyzed as previously described (75). In brief, thawed tissue sections were fixed and subjected to Molecular Cartography (100-plex combinatorial single-molecule fluorescence in situ hybridization) (Resolve Biosciences) using the manufacturer’s instructions (protocol 3.0). The probes were designed using Resolve’s proprietary design algorithm. Cell segmentation was performed following Resolve Biosciences’ pipeline (76, 77). After segmentation, we filtered the cell-wise gene expression data, retaining only cells with a minimum of 3 detected transcripts. These data were analyzed using the Seurat R package (v3), where SCTransform normalization was applied, followed by PCA using the RunPCA function and computation of shared nearest neighbor graph using FindNeighbors. Next the RunUMAP and FindClusters functions were used for data visualization and clustering, respectively. The 2D spatial component of the data was visualized by incorporation of centroid coordinates from the segmentation analysis.

### Primary human ileal fibroblasts and CCD-18Co colonic human fibroblasts.

Single-cell suspensions were directly cultured in a T-25 flask (90026, TPP) with RPMI 1640 medium (31870074, Gibco) and supplied with 10% FBS (BWSTS181H, VWR), 1% HEPES (15630056, Gibco), 1% l-glutamine (A2916801, Gibco), 1% sodium pyruvate (11360070, Gibco), and 1% antibiotic/antimycotic solution (A5955, Sigma-Aldrich). 0.25% trypsin-EDTA (25200056, Gibco) was used to detach the cells. Primary human ileal fibroblasts were obtained, purified as confirmed by use of vimentin (1:500; clone 280618, R&D Systems) and α-smooth muscle actin staining (1:500; NB300-978, Novus Biologicals) (data not shown), and used after 2 passages (78). CCD-18Co colonic fibroblasts (CRL-1459) were obtained from ATCC and cultured according to the ATCC culture guides.

For generation of CCD-18Co cells with stable *TWIST1* shRNA-mediated knockdown, lentiviral particles were generated using third-generation packaging vectors in human embryonic kidney (HEK) 293T cells using the MISSION pLKO.1-puro vector. Short hairpin RNA (shRNA) knockdown constructs for TWIST1 were obtained from the MISSION TRC shRNA library (Sigma-Aldrich) with the following sequences: TRCN0000020541, NM_000474.2-636s1c. As a non-targeting control, a non-human shRNA targeting sequence (SHC002, Sigma-Aldrich) was used. Cells were infected with viral particles overnight and subsequently selected/cultured with 2 μg/mL of puromycin (Sigma-Aldrich). To confirm TWIST1 knockdown, total RNA was isolated using the NucleoSpin RNA isolation kit (Macherey-Nagel) according to the manufacturer’s instructions. cDNA was synthesized with the RevertAid First Strand cDNA synthesis kit (Thermo Fisher Scientific). Quantitative reverse transcription PCR was performed with SYBR Green Master Mix (Bio-Rad Laboratories, Nazareth, Belgium) using the CFX96 Touch Real-Time PCR Detection System (Bio-Rad). Target genes were amplified using specific primers (Supplemental Methods).

Primary ileal fibroblasts and CCD-18Co fibroblasts were cultured in a 96-well plate for cell proliferation assay, scratch wound assay, in vitro ECM deposition, and immunofluorescent staining to determine fibroblast phenotypes and therapeutic function of harmine (5 μM; 286044, Sigma-Aldrich). Active concentrations of harmine were determined by IncuCyte proliferation assay (Sartorius AG) (data not shown). Different combinations of 5 ng/mL human IL-1α (R&D Systems), 5 ng/mL human IL-1β (R&D Systems), 5 ng/mL human TGF-β1 (R&D Systems), 5 ng/mL human TNF-α (R&D Systems), 5 ng/mL human IFN-γ (R&D Systems), or 5 ng/mL human OSM (R&D Systems) were used to stimulate fibroblasts along with 5% FBS RPMI 1640 medium.

### Statistics.

Data are shown as mean ± SEM. Multiple groups were compared by 1-way ANOVA or 2-way ANOVA with Tukey’s multiple-comparison test. Comparison between 2 groups was made using a 2-tailed Student’s *t* test. A *P* value of less than 0.05 was considered statistically significant. Statistical analysis of the data was performed using GraphPad Prism v9.1.0 software (GraphPad Inc.).

### Study approval.

The protocol for human specimens was approved by the Institutional Review Board (IRB) of University Hospitals Leuven, Belgium (B322201213950/S53684, CCARE, S-53684 and S64914). All recruitment was performed after ethical approval and oversight from the IRB, and written informed consent was obtained from all participants before surgery. Clinical information and metadata for the samples in this study are provided in Supplemental Methods. The protocol for animal studies was approved by the Animal Ethics Committee at KU Leuven (project 188/2019).

### Data availability.

Read count matrix of the scRNA-Seq is deposited under restricted access in the European Genome-phenome Archive (EGA; study submission number EGAS50000000382).

Requests for the data will be reviewed by the UZ Leuven–KU Leuven data access committee. Any data shared will be released via a data transfer agreement that will include the necessary conditions to guarantee protection of personal data (according to European General Data Protection Regulation law).

Any additional information required to reanalyze the data reported in this paper is available upon request.

## Author contributions

BJK, SA, BV, GDH, S Verstockt, and GM conceptualized the study. BJK, GD, SA, GB, VDS, PVdV, IDM, JF, GDH, and GM devised methodology. SA and SS provided software. BJK and SA performed formal analysis. BJK, SA, GZ, GD, FB, SS, AZ, LVB, VB, YVR, EV, S Verstockt, CB, SL, LJACH, BV, and GDH performed investigation. GB, AW, AD, GDH, SA, BV, S Vermeire, and GM provided resources. BJK, SA, and BV curated data. BJK and SA performed visualization. MS, EK, S Verstockt, and GM acquired funding. S Verstockt and GM performed project administration. S Verstockt and GM supervised the study. BJK and SA wrote the original draft. All authors reviewed and edited the manuscript.

## Supplementary Material

Supplemental data

Supporting data values

## Version 1 07/23/2024

In-Press Preview

## Version 2 08/15/2024

Electronic publication
