# Supplementary material for: Intercellular interaction between FAP+ fibroblasts and CD150+ inflammatory monocytes mediates fibrostenosis in Crohn’s disease
Source: J Clin Invest. 2024 Jul 23;134(16):e173835. doi: 10.1172/JCI173835 (PMC11324301; doi:10.1172/JCI173835)

Supplemental methods

FAP activity measurement

Snap-frozen ileum was crushed in a pre-cooled mortar kept on dry ice, intermittently using liquid nitrogen to keep samples frozen and to avoid loss of proteolytic activity due to temperature increase. Afterwards, the samples were lysed (1:5 ratio of sample (mg): lysis buffer (μL)) in lysis buffer (50 mM Tris-HCl pH 8.3, 10 mM EDTA, 1% n-octyl-β-D-glucoside and 70 μg/mL aprotinin) for 30 minutes on ice with frequent agitation. Next, samples were centrifuged at 12.000xg for 10 minutes at 4 °C. The ileum supernatant was collected and used immediately to perform a FAP enzymatic activity measurement and Bradford protein quantification assay. FAP enzymatic activity measurements were performed using the in-house developed assay using Z-Gly-Pro-AMC as the fluorogenic substrate and UAMC-1110 as specific FAP-inhibitor (1, 2).

Briefly, in a 96-well plate (half-area, Greiner Bio-One), 5 μL ileum supernatant was pre-incubated for 15 min at 37 °C with 10 μL of FAP inhibitor or solvent control (250 nM UAMC-1110 or 0.0025% (v:v) DMSO in FAP assay buffer consisting of 100 mM Tris-HCl pH 8.0, 300 mM NaF, 1 mM EDTA and 50 mM salicylic acid). Afterward, 35 μL pre-heated substrate solution (Z-Gly-Pro-AMC in FAP assay buffer, final concentration 266 μM; Bachem, Bübendorf, Switzerland, cat nr: 4002518) was added and fluorescence was measured kinetically for 30 minutes at 37 °C using the Tecan Infinite® M200 Pro (excitation wavelength 380 nm and emission wavelength 465 nm; Tecan, Männedorf, Switzerland). Fluorescence intensity was related to an AMC standard curve (0.3125 μM-10 μM) in an identical buffer. FAP enzymatic activity was normalised to the total protein content in the samples by a Bradford protein quantification assay.

Immunofluorescence staining of human ileum

The terminal ileum was fixed in 4% formaldehyde, embedded in paraffin and cut into 5μm thickness. Dewaxed and rehydrated 5 μm thick sections from the paraffin blocks s were subjected to heat-induced antigen retrieval by boiling in sodium citrate buffer (pH 6.0) for 20 minutes at 95°C (water bath). This was followed by 10 minutes of permeabilisation buffer (0.2% Triton X-100 in PBST) and 60 minutes of blocking in 2% BSA/PBST at room temperature. Tissue slides were labelled with primary antibodies in 2% BSA/PBST overnight at 4 °C, including sheep anti-human FAP antibody (1:40, AF3715, R&D Systems), rabbit anti-

human FAP antibody (1:50, clone E1V9V, Cell Signaling Technology), rabbit anti-human TWIST1 antibody (1:400, clone E7E2G, Cell Signaling Technology), mouse anti-human CD34 antibody (1:100, clone QBEnd/10, Sigma-Aldrich), rabbit anti-human ADAMDEC1 antibody (1:100, PA5-103574, Invitrogen), rabbit anti-human CD150 antibody (1:300, PA5-84809, Invitrogen), mouse anti-human CD68 antibody (1:100, clone KP1, BioLegend), and rat anti-human podoplanin antibody (1:300, clone NZ-1.3, eBioscience). Following incubation, the tissue slides were labelled with secondary antibodies in 2 % BSA/PBST for 2 hours at room temperature, including Alexa Fluor 488-conjugated donkey anti-sheep IgG antibody (A-11015, Invitrogen), Cy3-conjugated donkey anti-goat IgG antibody (705-165-003, Jackson ImmunoResearch), Cy3-conjugated donkey anti-rat IgG antibody (712-165-150, Jackson ImmunoResearch), Cy5-conjugated donkey anti-mouse IgG antibody (715-175-151, Jackson ImmunoResearch) and ATTO490LS-conjugated goat anti-rabbit IgG antibody (2309-1MG, Hypermol). Autofluorescence was removed by using Vector® TrueVIEW™ Autofluorescence Quenching Kit (SP-8400-1, Vector Laboratories).

Comparison of IBD data sets

For Seurat label transfer, we used our integrated mesenchymal data and myeloid data as references for the corresponding subsets of the 3 published IBD data sets (3–6). To transfer cell type labels, we employed the Seurat label transfer method. The "FindTransferAnchors" function was applied to identify anchors between the reference and each of the published datasets, utilising the CCA dimensionality reduction method. Subsequently, the "TransferData" function was used to transfer the labels from the reference dataset to the published datasets based on these anchors. Cell type classification probability was visualised with heatmaps.

To create a shared gene signature for FAP fibroblasts and Inflammatory monocytes in Inflammatory Bowel Disease (IBD), individual datasets were divided into two groups: myeloid cells (monocytes, macrophages, and dendritic cells) and mesenchymal cells (fibroblasts, myofibroblasts, and pericytes). The data were then analysed to identify clusters of cells that were similar to specific cell types such as FAP fibroblasts or Inflammatory monocytes. Similarity was established based on Seurat label transfer (Figure S2I,). These subsets were annotated as Inflammatory fibroblasts/Activated fibroblasts or Inflammatory monocytes in various studies. Fibroblast subsets similar to FAP fibroblast were annotated as *Inflammatory fibroblasts* in Smillie et al., *activated fibroblasts* in Martin et al. and *Inflammatory fibroblasts IL11 CHI3L1* in Kong et al. Myeloid subsets similar to inflammatory monocytes were as

annotated as *Inflammatory monocytes* in Smillie et al., *Inflammatory macrophages* in Martin et al. and *Monocytes S100A8 S100A9* in Kong et al.

Next, significantly upregulated genes (with an adjusted p-value <0.05) in the Inflammatory monocytes or Inflammatory fibroblasts were identified within the respective compartments of each dataset using the FindMarkers function from Seurat. The genes common in all these lists of upregulated genes in inflammatory fibroblasts in each data set formed the common gene signature for Inflammatory monocytes or FAP fibroblasts in IBD (Supplemental data). Finally, a single Seurat object with raw counts was created for each cell compartment (myeloid or mesenchymal), retaining the original annotation and meta-data, and normalised. The gene signature was then scored for each cell compartment using the AddModuleScore function from Seurat.

RNA isolation and gene expression

RNA was extracted utilising the micro plus Kit (Qiagen), following the manufacturer's guidelines. cDNA was then synthesised the High-Capacity cDNA Reverse Transcription Kit (ThermoFisher Scientific), as per the provided instructions. The RT-PCR process was carried out using the LightCycler 480 SYBR Green I Master (Roche) on a Light Cycler 480 instrument (Roche). The $2^{-\Delta CT}$ method was employed to quantify the results, and gene expression levels of interest were normalised relative to the reference gene *RPLP0*.

Protein quantification

Supernatants from sorted myeloid cells were processed as described above. The Meso Scale Discovery (MSD) ELISA-based assay platform employed U-PLEX Custom Biomarker (hu) Assays (K15067M-1, LOT 473953) for quantifying protein concentrations of IL-1α, IL-1β, IFNγ, and TNFα, following the manufacturer's manual. Additionally, the U-PLEX Human TGF-β1 Assay (K151XWK-1, LOT 473954) was utilized to quantify the protein concentration of TGF-β1. The analyses were conducted using a 4PL fitting method to calculate concentrations.

***In vitro* immunofluorescence staining**

CCD18-Co colonic human fibroblast (CRL-1459, ATCC) or primary ileal fibroblasts (10,000 cells per well) were cultured in the matrix-gel-coated PhenoPlate™ 96-well microplates (PerkinElmer) with inflammatory cytokines or myeloid derived supernatants along with Harmine (5 µM, 286044, Sigma-Aldrich) for 24 or 48 hours. Then the cells were fixed with ice-cold acetone (Chem-Lab) and labelled with sheep anti-human FAP antibody (1:40,

AF3715, R&D Systems), mouse anti-human TWIST1 antibody (1:100, clone 2F8E7, Invitrogen), goat anti-human collagen III alpha 1 antibody (1:500, NBP1-26547, Novus Biologicals), ATTO 490LS-conjugated phalloidin (1:200, 14479, Sigma-Aldrich), Alexa Fluor 488-conjugated donkey anti-sheep IgG antibody (A-11015, Invitrogen), Cy3-conjugated donkey anti-goat IgG antibody (705-165-003, Jackson ImmunoResearch) and Cy5-conjugated donkey anti-mouse IgG antibody (715-175-151, Jackson ImmunoResearch). Immunofluorescent staining was imaged on the Operetta CLS high-content analysis system (PerkinElmer) and analysed on Harmony 4.5 software (PerkinElmer) at the VIB Leuven.

***In vitro*-extracellular matrix (ECM) deposition**

To generate fibroblast-derived ECM, we modified the protocol of Kaukonen et al (7). Briefly, 0.2% (wt/vol) gelatin (G6144, Sigma-Aldrich)-coated PhenoPlate™ 96-well microplates (PerkinElmer) were treated with 1% (vol/vol) glutaraldehyde (G5882, Sigma-Aldrich) to create cross-link, following by 1 M glycine (1.04201, Millipore) to aspirate the cross-linker. CCD18-Co colonic human fibroblast (CRL-1459, ATCC) or primary ileal fibroblasts (30,000 cells per well) were seeded until 85-90% confluence. Fibroblasts were treated with/without inflammatory cytokines or with/without myeloid derived supernatants, described above for 72 hours, following by 50 μg/mL L-ascorbic acid (A4544, Sigma-Aldrich) treatment to promote collagen cross-linking daily. Fibroblasts were re-stimulated to reboot fibrotic phenotype every seven days. This cycle was repeated for three weeks till ECM was well deposited. Harmine (5 μM, 286044, Sigma-Aldrich) were given and refreshed daily after first stimulation.

ECM was labelled with primary antibodies, including goat anti-human collagen III alpha 1 antibody (1:500, NBP1-26547, Novus Biologicals), rabbit anti-human collagen I antibody (1:500, NB600-408, Novus Biologicals), mouse anti-human collagen IV antibody (1:500, clone 1042, eBioscience), sheep anti-human FAP antibody (1:40, AF3715, R&D Systems), and sheep anti-human fibronectin antibody (1:500, AF1918, R&D Systems) in cell culture medium for 1 hour in the cell culture incubator. Then the cells were labelled with secondary antibodies, including Alexa Fluor 488-conjugated donkey anti-mouse IgG antibody (715-545-150, Jackson ImmunoResearch), Cy3-conjugated donkey anti-goat IgG antibody (705-165-003, Jackson ImmunoResearch), Cy3-conjugated donkey anti-rabbit IgG antibody (711-165-152, Jackson ImmunoResearch) and Alexa Fluor 647-conjugated donkey anti-sheep IgG antibody (713-605-147, Jackson ImmunoResearch) before 4 % paraformaldehyde fixation (1040051000, Merck). ECM deposition was imaged on the LSM780 confocal microscope

(Zeiss) with multiple focal planes (Z-stacks) and on the Operetta CLS high-content analysis system (PerkinElmer) and analysed on Harmony 4.5 software (PerkinElmer).

Human-induced pluripotent stem cells (iPSCs) maintenance

Human-induced pluripotent stem cells (iPSCs) (WiCell, DF19-9-7T) were cultured on Cultrex Extract (3434-001-02, Stem Cell Qualified Reduced Growth Factor Basement Membrane, R&D Systems)-coated tissue culture dishes and were maintained in the mTeSR™ Plus medium (100-0274, STEMCELL Technologies). The medium was changed every two days. When the cells reached about 80 % confluency, they were passaged using TrypLE™ Express Enzyme (12604013, Gibco), and the 10 µM RHO/ROCK pathway inhibitor (129830-38-2, STEMCELL Technologies) was used for 24 hours. The next day, the cells were washed with fresh medium and maintained until the next passage.

Intestinal organoids (IOs) differentiation

Human intestinal organoids (IOs) were differentiated from iPSCs using an adapted protocol already published (8, 9). The iPSCs were induced into 3D IOs in a three-step protocol. Briefly, on day 0, confluent iPSCs were induced into definitive endoderm (DE) differentiation using RPMI-1640 (31870074, Gibco) supplemented with Activin A (100 ng/mL, 338-AC, R&D Systems). The next day, the medium was changed and fresh RPMI-1640 (31870074, Gibco) supplemented with Activin A (100 ng/mL, 338-AC, R&D Systems) and 0.2% HyClone-defined FBS (GE Healthcare Bio-Sciences) was added. On the 3rd and 4th days, the medium was changed and RMPI-1640 (31870074, Gibco) supplemented with Activin A (100 ng/mL, 338-AC, R&D Systems) and 2 % HyClone-defined FBS (GE Healthcare Bio-Sciences) was added. Further, the DE was induced to mid- and hindgut differentiation by changing the medium daily (RPMI-1640 supplemented with 15 mM HEPES, 2% HyClone-defined FBS, FGF-4 (500 ng/mL, 235-F4, R&D Systems), WNT-3a (500 ng/mL, 5036-WN, R&D Systems) until the 3D spheroids were formed. Spheroids were collected and embedded in a drop Cultrex RGF Basement Membrane Extract, Type 2 (3536-005-02, R&D Systems) and fed with IOs complete medium (Advanced DMEM F12 (1264-010, Gibco) supplemented with B27 supplement (17504-044, Thermo Fisher Scientific), GluMAX supplement (35050-038, Thermo Fisher Scientific), penicillin and streptomycin (500 U/mL), R-spondin 1 (500 ng/mL, 4645-RS, R&D Systems), Noggin (100 ng/mL, 6057-NG, R&D Systems), EGF (100 ng/mL, 236-EG,R&D Systems). The medium was changed twice a week. After 50 days the IOs were used for the experiments.

Immunofluorescence staining of IOs

IOs were washed with PBS and fixed with 4 % paraformaldehyde (PFA) for 20 minutes at room temperature and then washed three times with PBS. The IOs were dehydrated in 15 % sucrose at 4°C overnight. The IOs were frozen in Tissue Freezing Medium (Leica Biosystems). The samples were cut and rehydrated in PBS for 10 minutes. Subsequently, the samples were blocked with 1 % bovine serum albumin (BSA) at room temperature for 45 minutes and incubated with primary anti-FAP (1:200, F11-24, eBioscience), anti-PDPN (1:100, NZ-1.3, eBioscience), and anti-E-Cadherin (1:300, 24E10, Cell Signaling Technology) antibodies at 4 °C overnight. The samples were washed three times with PBS and then incubated with secondary antibodies, including Alexa Fluor 555-conjugated goat anti-mouse IgG antibody (A32727, Invitrogen), Alexa Fluor 488-conjugated donkey anti-rat IgG antibody (A-21208, Invitrogen), and Alexa Fluor 647-conjugated goat anti-rabbit IgG antibody (A-21245, Invitrogen) for one hour at room temperature. The samples were washed with PBS three times and stained with DAPI for twenty minutes at room temperature. The samples were washed with deionised water and embedded in Mowiol. The samples were acquired with Zeiss LSM 780 confocal microscope with 10x magnification and analysed with Fiji ImageJ.

Chronic DSS colitis

Animal studies (project P188/2019) were performed after approval by the KU Leuven Ethical Committee for Animal Experimentation. Mice (aged 10 weeks) used for this study were group-housed under controlled temperature (22°C) and photoperiod (12:12-hour light-dark cycles) conditions and given unrestricted access to standard rodent diet and water (or DSS drinking solution). The mice were subjected to 3 cycles of 2% DSS (w/v) (40 kDa, DS001, TdB Labs) administration (7 days of DSS administration followed by 14 days of regular drinking water was defined as one cycle). The mice were monitored for signs of colitis each day (i.e., body weight loss, diarrhoea score and blood in the faeces). Hemoccult II® Fecal Occult Blood Test Kit (61200, Beckman Coulter) was used to detect blood in faeces.

B6;129S7-$Twist1^{tm2Bhr}$/Mmnc mice were acquired from the Mutant Mouse Resource and Research Center (MMRRC_016842-UNC) (10). The COL1A2$^{Cre\text{-}ER}$ mice were acquired from the Jackson Laboratory (IMSR_JAX:029567) (11). TWIST1$^{\Delta/\Delta COL1A2}$ mice were generated by crossbreeding from $Twist1^{fl/fl}$ mice and COL1A2$^{Cre\text{-}ER}$ mice. Genotype were confirmed by PCR with the following sequence primer shown in Supplemental data. Tamoxifen (3mg/mouse/day, T5648-5G, Sigma) in sesame seed oil was oral gavaged to

Twist1$^{\Delta/\Delta Col1a2}$ mice and littermates daily for 5 consecutive days one week prior of every DSS cycle to induce Cre activity. Male wild type C57BL/6J mice were aquired from Envigo (Harlan) for evaluating anti-fibrotic properties of Harmine. Harmine (10 mg/kg) was dissolved in DMSO and Tween-80 and injected intraperitoneally from day 0 to day 10 during each DSS cycle. The groups without Harmine treatment received DMSO and Tween-80 intraperitoneally. Mice were euthanised using CO^2 and colons collected for further analysis.

Picrosirius red staining of the mouse colons

Mouse colon was fixed in 4% formaldehyde, and biopsies were embedded in paraffin and cut into 5µm thick sections for histological analysis. In brief, the tissue slides were de-waxed by HistoChoice clearing agent (H2779-1L, Sigma-Aldrich) and rehydrated by the gradient decrease of ethanol solution and water. Then the tissue was stained in 0.1% of Direct Red 80 (365548-5G, Sigma-Aldrich) in picric acid solution (P6744-1GA, Sigma-Aldrich) for an hour, followed by two changes of acidified water and 100% ethanol. To quantify the relative histologic area of collagen on Masson's trichrome stained slides, we took an average of ten images in each sample on Leica DM2500 M and quantified them with Image J.

Histology score of mouse colon

A haematoxylin and eosin (H&E) staining was performed to determine the rate of damage and inflammation in the colon. Each section was blindly scored using a validated scoring system (12). Briefly, samples were checked for submucosal infiltration (0; none up to 3; large infiltrate), goblet cell loss (0; none up to 3; >50%), crypt density (0; normal up to 3; decreased by >50%) and crypt hyperplasia (0; none up to 3; >3-fold increase in crypt length). The total score was calculated as 1x Goblet cell loss + 2x Crypt Density + 2x Hyperplasia + 3x Submucosal infiltrate and it is reflected in the Mouse Colitis Histology Index (MHCI).

Immunofluorescence staining of mouse colon

The distal colon (0.5 mm away from the proximal colon) was fixed in 4% formaldehyde overnight, embedded in paraffin and cut into 5µm thickness. Dewaxed and rehydrated 5 µm thick sections from the paraffin blocks s were subjected to heat-induced antigen retrieval by boiling in sodium citrate buffer (pH 6.0) for 20 minutes at 95°C (water bath). This was followed by 10 minutes of permeabilisation buffer (0.2% Triton X-100 in PBST) and 60 minutes of blocking in 2% BSA/PBST at room temperature. Tissue slides were labelled with primary antibodies in 2% BSA/PBST overnight at 4 °C, including sheep anti-human FAP antibody (1:40, AF3715, R&D Systems), and rabbit anti-mouse TWIST1 antibody (1:250, clone

E2M5V, Cell Signaling Technology). Following incubation, the tissue slides were labelled with secondary antibodies in 2 % BSA/PBST for 2 hours at room temperature, including Alexa Fluor 488-conjugated donkey anti-sheep IgG antibody (A-11015, Invitrogen), Alexa Fluor 647-conjugated AffiniPure™ Fab Fragment Donkey Anti-Rabbit IgG (H+L) (711-607-003, Jackson ImmunoResearch) and DAPI. Autofluorescence was removed by using Vector® TrueVIEW™ Autofluorescence Quenching Kit (SP-8400-1, Vector Laboratories).

Primary mouse colonic fibroblasts from B6;129S7-*Twist1^{tm2Bhr}*/Mmnc mice

Mouse colons were treated with 1 mM DTT and 1 mM EDTA in 1x Hank's balanced salt solution (HBSS), and 1 mM EDTA in HBSS at 37 °C for 15 minutes, respectively. Then the tissue was minced and digested with 2.7 U/mL collagenase D (Roche Applied Science), 50 U/mL DNase I (Sigma), and 19.8 U/mL dispase II (Gibco) in a sterile gentleMACS C tube for 20 minutes at 37 °C at 250 to 300 rpm after dissociating with the gentleMACS™ Dissociator (program human_tumor_02.01). After being treated with Red Blood Cell Lysis Buffer (11814389001, Roche), single-cell suspensions were directly cultured in a T-25 flask (90026, TPP) with RPMI-1640 medium (31870074, Gibco), supplied with 10% FBS (BWSTS181H, VWR), 1% HEPES (15630056, Gibco), 1% L-glutamine (A2916801, Gibco), 1% sodium pyruvate (11360070, Gibco), and 1 % antibiotic/antimycotic solution (A5955, Sigma-Aldrich). 0.25% Trypsin-EDTA (25200056, Gibco) was used to detach the cells. Primary mouse colonic fibroblasts were obtained, purified confirmed by using vimentin (1:500, clone 280618, R&D systems) and alpha-smooth muscle actin staining (1:500, NB300-978, Novus Biologicals) (data not shown) and used after two passages (13). TWIST1 depletion was induced by TAT-CRE Recombinase (SCR508, Sigma-Aldrich) following the manufacturer's standard protocol and confirmed by Purified anti-Cre Recombinase Antibody (908002, BioLegend). The fibroblast stimulation and immunofluorescence staining procedures are identical to those detailed above.

Reference

1. Bracke A, et al. The development and validation of a combined kinetic fluorometric activity assay for fibroblast activation protein alpha and prolyl oligopeptidase in plasma. *Clinica Chimica Acta*. 2019;495:154–160.

2. Jansen K, et al. Extended structure-activity relationship and pharmacokinetic investigation of (4-quinolinoyl)glycyl-2-cyanopyrrolidine inhibitors of fibroblast activation protein (FAP). *J Med Chem*. 2014;57(7):3053–3074.

3. Stuart T, et al. Comprehensive Integration of Single-Cell Data. *Cell*. 2019;177(7):1888-1902.e21.
4. Smillie CS, et al. Intra- and Inter-cellular Rewiring of the Human Colon during Ulcerative Colitis. *Cell*. 2019;178(3):714-730.e22.
5. Martin JC, et al. Single-Cell Analysis of Crohn's Disease Lesions Identifies a Pathogenic Cellular Module Associated with Resistance to Anti-TNF Therapy. *Cell*. 2019;178(6):1493-1508.e20.
6. Kong L, et al. The landscape of immune dysregulation in Crohn's disease revealed through single-cell transcriptomic profiling in the ileum and colon. *Immunity*. 2023;56(2):444-458.e5.
7. Kaukonen R, et al. Cell-derived matrices for studying cell proliferation and directional migration in a complex 3D microenvironment. *Nat Protoc*. 2017;12(11):2376–2390.
8. McCracken KW, et al. Generating human intestinal tissue from pluripotent stem cells in vitro. *Nat Protoc*. 2011;6(12). https://doi.org/10.1038/nprot.2011.410.
9. Jose SS, et al. Comparison of two human organoid models of lung and intestinal inflammation reveals Toll-like receptor signalling activation and monocyte recruitment. *Clin Transl Immunology*. 2020;9(5). https://doi.org/10.1002/cti2.1131.
10. Chen Y-T, et al. Generation of a Twist1 conditional null allele in the mouse. *genesis*. 2007;45(9):588–592.
11. Zheng B, et al. Ligand-Dependent Genetic Recombination in Fibroblasts: A Potentially Powerful Technique for Investigating Gene Function in Fibrosis. *Am J Pathol*. 2002;160(5):1609–1617.
12. Koelink PJ, et al. Development of Reliable, Valid and Responsive Scoring Systems for Endoscopy and Histology in Animal Models for Inflammatory Bowel Disease. *J Crohns Colitis*. 2018;12(7):794–803.
13. Johnson P, et al. Isolation of CD 90+ fibroblast/myofibroblasts from human frozen gastrointestinal specimens. *Journal of Visualized Experiments*. 2016;2016(107). https://doi.org/10.3791/53691.

Supplemental figures

Figure S1. Single-cell profiling of fibro-stenotic ileal from CD and control ileum from CRC, related to Figure 1. (**A**) H&E and Masson's trichrome stain showing signs of inflammation and fibrosis. (**B**) the plot of histologic scores and collagen quantification in different lesions of terminal ileum. Data are shown as box and whisker plots. Statistically significant differences were determined using a one-way ANOVA test corrected with Tukey's multiple comparisons test (*p <0.05, ** p <0.01, *** p <0.005, ****p <0.001). (**C**) Pie plot showing proportions of cells from different lesions of terminal ileum. (**D**) Bar plot showing cell proportions in different lesions in CD's terminal ileum. (**E**) Bar plot showing percentage of cell clusters among the patients. (**F**) Heatmap showing core ECM genes in each cell type.

Figure S2. Heterogeneity of fibroblast in fibro-stenotic CD, related to Figure 2. (**A**) Bar plot showing proportions of mesenchymal subtypes in each terminal ileal lesion sample from each CD patient in the scRNA-seq data. (**B**) Contour plot showing gating strategy of fibroblast subsets by flow cytometry. (**C**) and (**D**) Percentage of fibroblast subsets in different lesions of terminal ileum are shown as bar plot with SEM. Statistically significant differences were determined using a one-way ANOVA test corrected with Tukey's multiple comparisons test (*p <0.05, **p <0.01). (**E**) Before-after plot showing FAP activity in different lesions of terminal ileum. Data is shown as a before-after plot. Statistically significant differences were determined using a one-way ANOVA test corrected with Tukey's multiple comparisons test (*p <0.05, **p <0.01). (**F**) Immunofluorescence staining for PDPN, ADAMDEC1, CD34 and FAP expression in healthy ileum and CD diseased ileum. Original image composed of stitched 25× images. (**G**) Heatmap depicting expression of chemokine and chemokine ligand genes in fibroblast subsets. (**H**) ssGSEA scores depicted as ridgeplots for selected terms, related to inflammation in fibroblast subsets. (**I**) Cross-dataset cell type prediction score heatmap showed similarity of stromal cell subset among published human IBD atlas. (**J**) Contour plot showing gating strategy of fibroblast subsets in colon by flow cytometry. (**K**) Percentage of fibroblast subsets in different lesions of colon from CRC (n=3), CD (n=3) and UC patients (n=4) are shown as bar plot with SEM. Statistically significant differences were determined using a one-way ANOVA test corrected with Tukey's multiple comparisons test (***p <0.005). (**L**) ssGSEA scores depicted as ridgeplots for selected terms, related to EMT in fibroblast subsets. (**M**) ssGSEA scores depicted as ridgeplots for selected terms, related to cellular senescence in fibroblast subsets. (**N**) Heatmap showing relative expression of senescence-associated secretory phenotype (SASP) markers.

Figure S3. Fibroblast-myeloid cell interaction modulates fibro-stenosis, related to Figure 3. (**A**) Partition-based graph abstraction (PAGA) analysis on fibroblast subsets showing most likely trajectories. (**B**) Heatmap showing gene expression change along cell Monocle3 pseudo-time. (**C**) Cellphone DB dot plot showing ligand-receptor interactions between mesenchymal compartment and immune cells. First and second interacting molecules correspond to first and second cell types on the y axis respectively. Black circles indicate significant interactions (**D**) ssGSEA scores depicted as ridgeplots for selected terms in fibroblast subsets.

Figure S4. Heterogeneity of myeloid cells in fibro-stenotic CD, related to Figure 4. (A) Bar plot showing proportion of myeloid subsets in each terminal ileal lesion sample from each CD patient in the scRNA-seq data. (**B**) Selected terms from the Reactome biological pathway enrichment analysis for differentially upregulated genes in Inflammatory monocytes (logFC,>0.5; FDR, <0.1). (**C**) Heatmap showing number of interactions (Ligand-Receptor pairs) between myeloid and mesenchymal. (**D**) SCENIC showing relative transcription factor activity in each myeloid cell subset. (**E**) and (**F**) Pseudo-time trajectory projected onto a UMAP of selected myeloid subsets. (**G**) Contour plot showing gating strategy of myeloid subsets by flow cytometry among the different disease states. (**H**) Bar plots showing cell proportion of myeloid subsets in different lesions of terminal ileum. Data are shown as bar plots with SEM. Statistically significant differences were determined using a one-way ANOVA test corrected with Tukey's multiple comparisons test (*p <0.05, ** p <0.01, *** p <0.005, **** p <0.001). (**I**) Immunofluorescence staining for CD68, SLAMF1 (CD150) and FAP expression in healthy ileum and CD diseased ileum. Original image composed of stitched 25× images. (**J**) Cross-dataset cell type prediction score heatmap showed similarity of myeloid cell subset among published human IBD atlas. (**K**) Contour plot showing gating strategy of myeloid subsets by flow cytometry among the colon from CRC (n=3), CD (n=3) and UC patients (n=4). (**L**) Bar plots showing cell proportion of CCR2 monocytes and CD150 Inflammatory monocytes in different lesions of colon. Data are shown as bar plots with SEM. Statistically significant differences were determined using a one-way ANOVA test corrected with Tukey's multiple comparisons test (**p <0.01, *** p <0.005, **** p <0.001). (**M**) Bar plots showing $CD150^-$ monocytes and $CD150^+$ monocytes in PBMC of healthy donor and CD patients. Data are shown as mean with SEM. Statistically significant differences were determined using a t-test.

Figure S5. Gene expression of FAP fibroblasts and Inflammatory monocytes in inflamed and stenotic ileum of fibro-stenotic CD patients, related to Figure 5. (**A**) Heatmap showing gene expression of cell clusters based on Molecular Cartography of transmural CD ileum (n=3).

Figure S6. CD150^{+} monocytes-derived cytokines promote FAP fibroblast activation and extra-cellular matrix protein deposition, related to Figure 6. (**A**) Experimental workflow showing FACS-sorting gating strategy and setup for *in vitro* ECM production. (**B**) Bar plot showing the protein expression of IL-1α, IL-1β, IFNγ, TNFα and TGF-β1. Data are shown as bar plots with SEM. Statistically significant differences were determined using a one-way ANOVA test corrected with Tukey's multiple comparisons test (**p* <0.05, ** *p* <0.01, *** *p* <0.005, **** *p* <0.001). (**C**) Heatmap showing relative expression of FAP, TWIST1 and type III collagen in CCD-18Co fibroblast treated with supernatant from FACS sorted myeloid subsets (n= 5 individual patients). (**D**) Experimental workflow. (**E**) Heatmap showing extracellular deposition of FAP, type I collagen and type III collagen in monocyte-stimulated CCD-18Co fibroblasts. (**F**) Heatmap showing relative protein expression of FAP, TWIST1 and type II collagen in primary ileal fibroblasts after being stimulated by selected cytokine combinations, predicted by NicheNet. (**G**) Experimental workflow. (**H**) Immunofluorescence staining (10× image) and (**I**) Bar plot showing relative expression of fibronectin and type IV collagen in *pro-fibrotic cues*-stimulated primary ileal fibroblasts after TWIST1 inhibition. Data are shown as bar plots with SEM. Statistically significant differences were determined using a one-way ANOVA test corrected with Tukey's multiple comparisons test (**p* <0.05, ** *p* <0.01, *** *p* <0.005, **** *p* <0.001).

Figure S7. TWIST1 inhibition attenuates intestinal fibrosis, related to Figure 7. X-Y plots showing (**A**) disease activity index (DAI) (**B**) body weight change (**C**) stool blood score and (**D**) stool consistency through chronic DSS colitis in $Twist1^{\Delta/\Delta Col1a2}$ mice. Data are shown as mean with SEM. Statistically significant differences were determined using a two-way ANOVA test. (**E**) Picrosirius red staining and (**F**) Violin plot showing collagen deposition in mouse colon of chronic DSS colitis in $Twist1^{\Delta/\Delta Col1a2}$ mice. Data are shown as mean with SEM. Statistically significant differences were determined using a t-test. (**G**) Violin plot showing colon tissue size post chronic DSS colitis in $Twist1^{\Delta/\Delta Col1a2}$ mice. Data are shown as mean with SEM. Statistically significant differences were determined using a t-test. Violin plot showing (**H**) mouse colitis histology index (**I**) goblet cell loss score (**J**) crypt density (**K**) crypt

hyperplasia and (**L**) submucosal infiltrate in mouse colon of chronic DSS colitis in *Twist1*$^{Δ/ΔCol1a2}$ mice. Data are shown as mean with SEM. Statistically significant differences were determined using a t-test. (**M**) Immunofluorescence staining (10× image) showing FAP expression in colon of *Twist1*$^{Δ/ΔCol1a2}$ mice post chronic DSS colitis. The scale bar represents a distance of 250 µm. (**N**) Violin plot showing median fluorescence intensity of FAP in stromal cells in colon of *Twist1*$^{Δ/ΔCol1a2}$ mice post-acute DSS colitis. Data are shown as mean with SEM. Statistically significant differences were determined using a t-test. (**O**) Immunofluorescence staining (25× image) and (**P**) Heatmap showing relative expression of FAP, TWIST1, type III and type IV collagen in *pro-fibrotic cues*-stimulated TWIST1 knockout fibroblasts from mouse. Data are shown as bar plot with SEM. The scale bar represents a distance of 100 µm. (**Q**) Picrosirius red staining and **(R)** Bar plot showing collagen deposition in mouse colon of chronic DSS colitis after Harmine treatment. Data are shown as bar plots with SEM. Statistically significant differences were determined using a one-way ANOVA test corrected with Tukey's multiple comparisons test (*p <0.05, **p <0.01). (**S**) Bar plot showing mouse colitis histology index. Data are shown as bar plots with SEM. Statistically significant differences were determined using a one-way ANOVA test corrected with Tukey's multiple comparisons test (*p <0.05, **p <0.01). (**T**) Immunofluorescence staining for FAP and TWIST1 expression in mouse colon of chronic DSS colitis after Harmine treatment. Original image composed of stitched 25× images. The scale bar represents a distance of 100 µm. X-Y plots showing (**U**) disease activity index changing, (**V**) body weight change, (**W**) stool blood score and (**X**) stool consistency through chronic DSS colitis with/without Harmine treatment. Data are shown as X-Y plots with SEM. Statistically significant differences were determined using a one-way ANOVA test corrected with Tukey's multiple comparisons test (*p <0.05, ** p <0.01, *** p <0.005, **** p <0.001).

Figure S8. Previously published datasets using mucosal biopsies do not show consistent presence of FAP fibroblasts and inflammatory monocytes across IBD patients, related to Figure S2 and S4. (**A**) Dotplot showing gene signature score for common markers of inflammatory fibroblasts in 4 independent IBD data sets. The area between the two horizontal lines indicates the interquantile range of the gene signature score for the cells in Ke & Abdurahiman et al. (**B**) Dotplot showing gene signature score for common markers of inflammatory monocytes in 4 independent IBD data sets. The area between the two horizontal lines indicates the interquantile range of the gene signature scores for the cells in Ke & Abdurahiman et al.

Figure S1. Single-cell profiling of fibro-stenotic ileal from CD and control ileum from CRC, related to Figure 1.

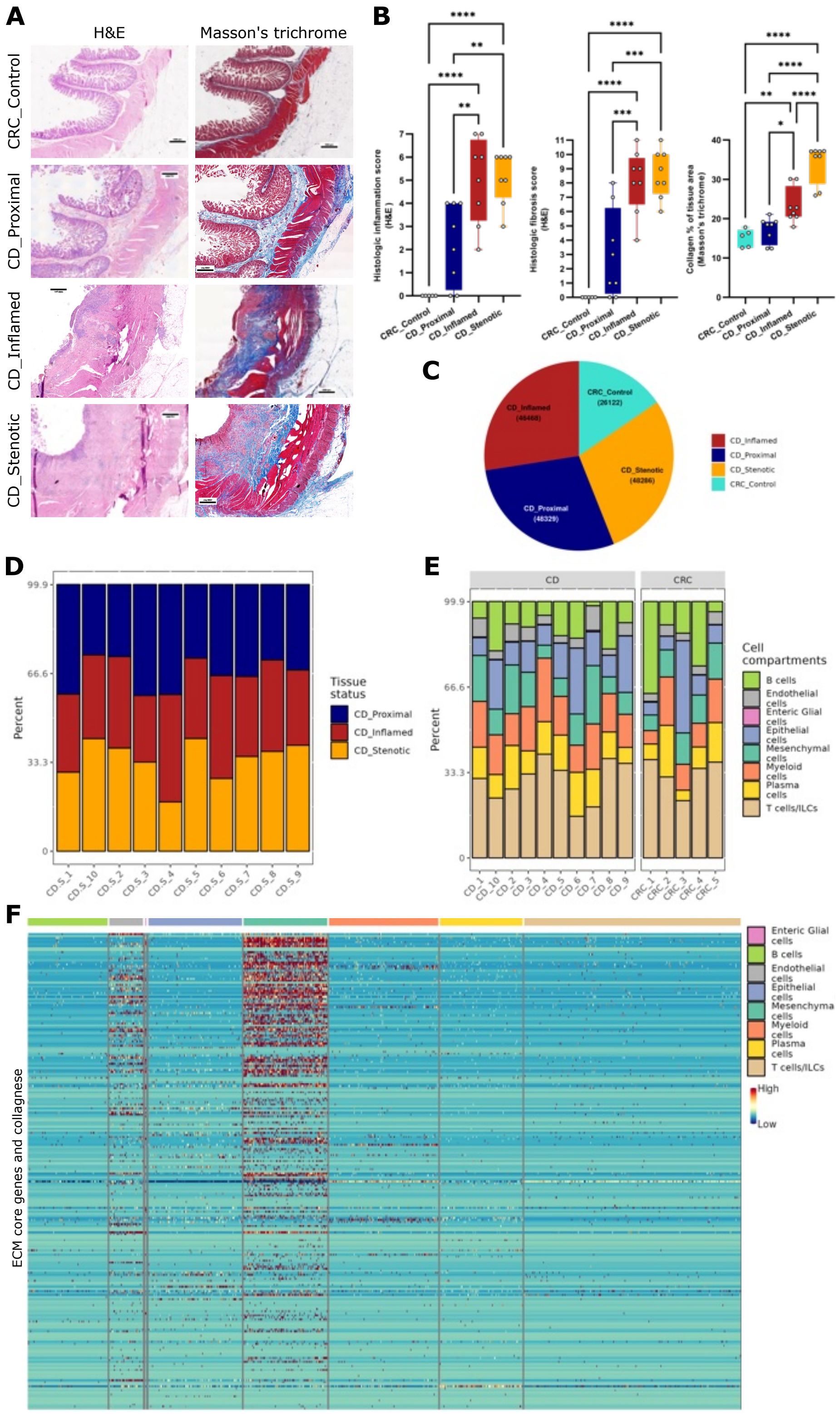

Figure S2. Heterogeneity of fibroblast in fibro-stenotic CD, related to Figure 2.

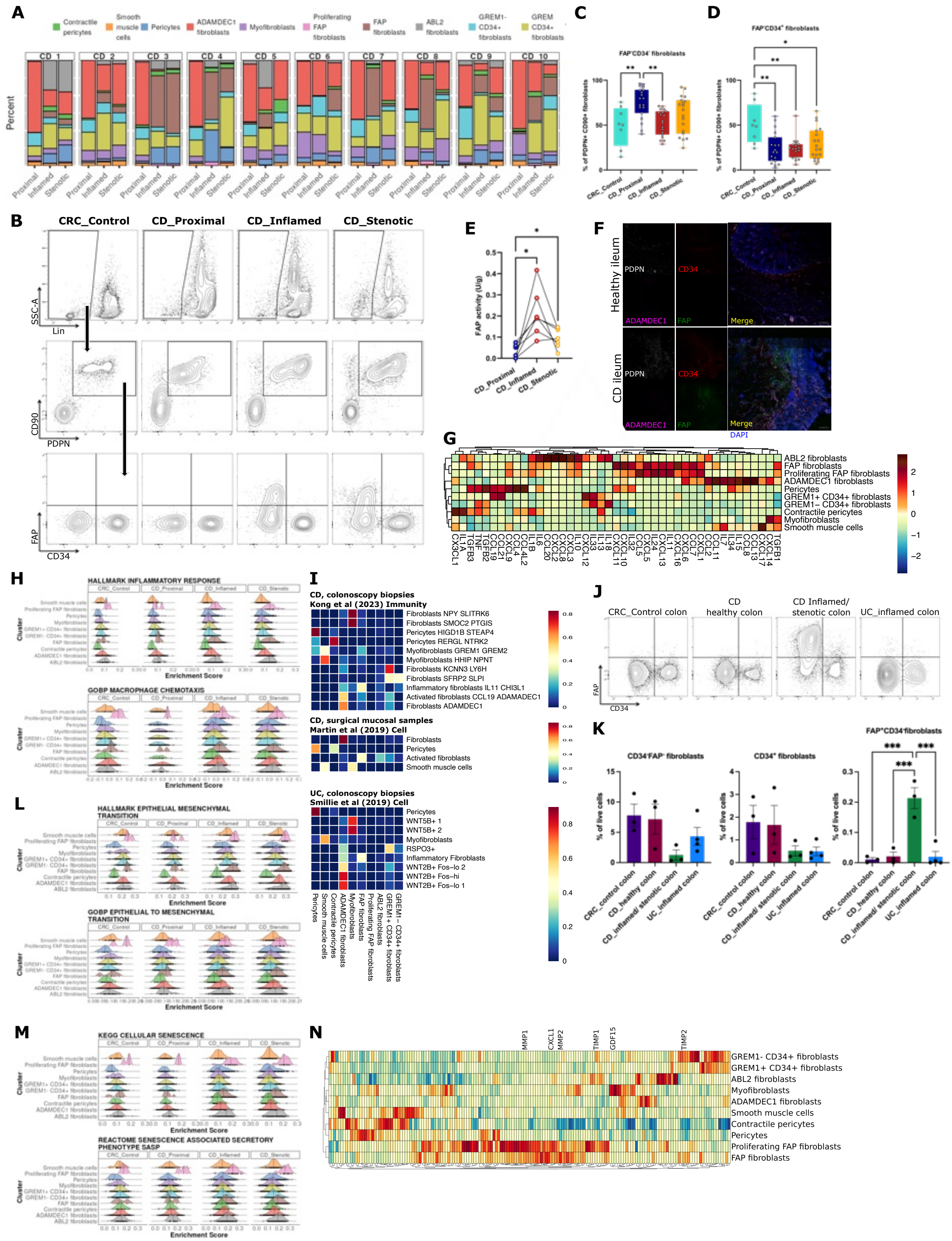

Figure S3. Fibroblast-myeloid cell interaction modulates fibro-stenosis, related to Figure 3.

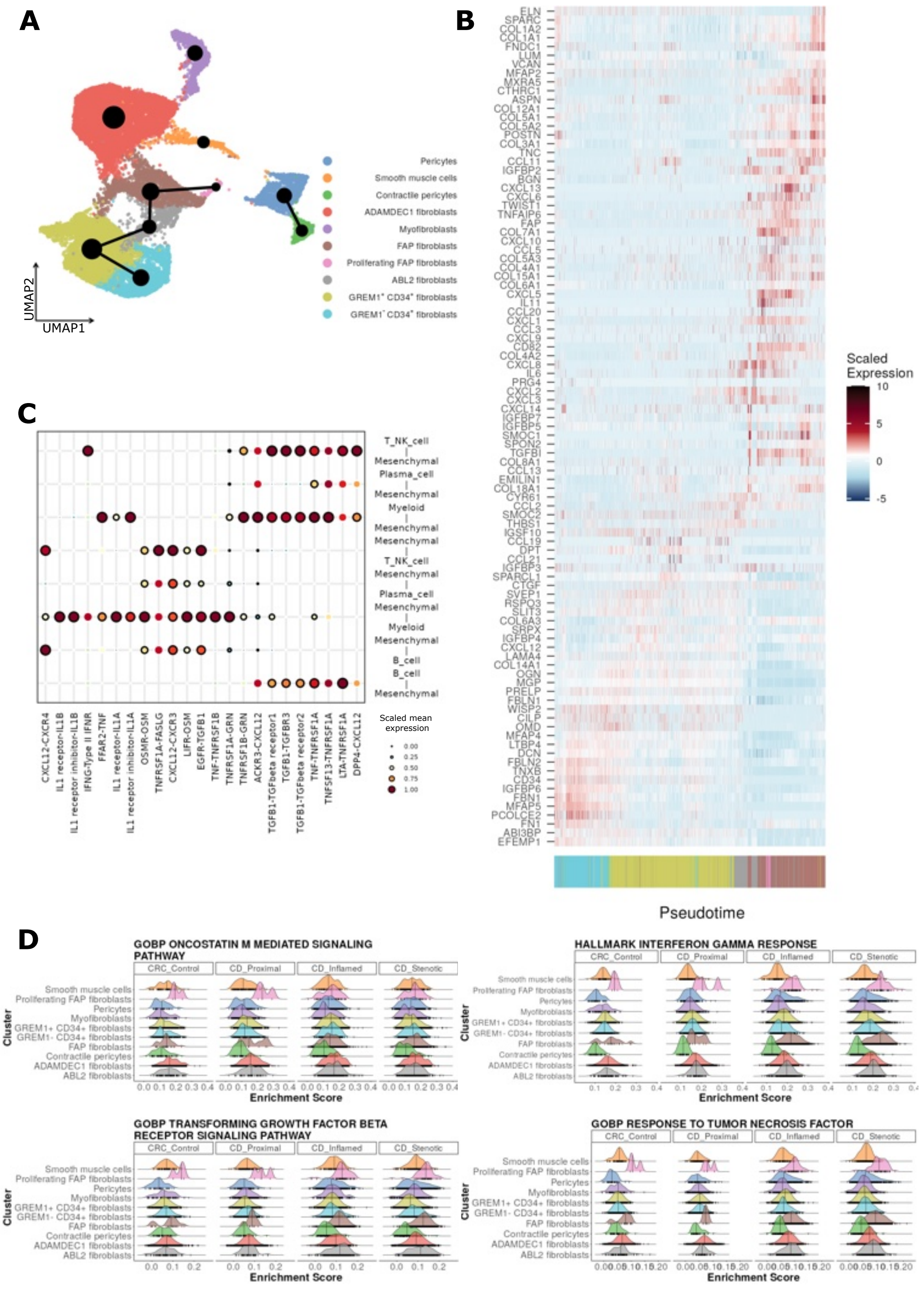

Figure S4. Heterogeneity of myeloid cells in fibro-stenotic CD, related to Figure 4.

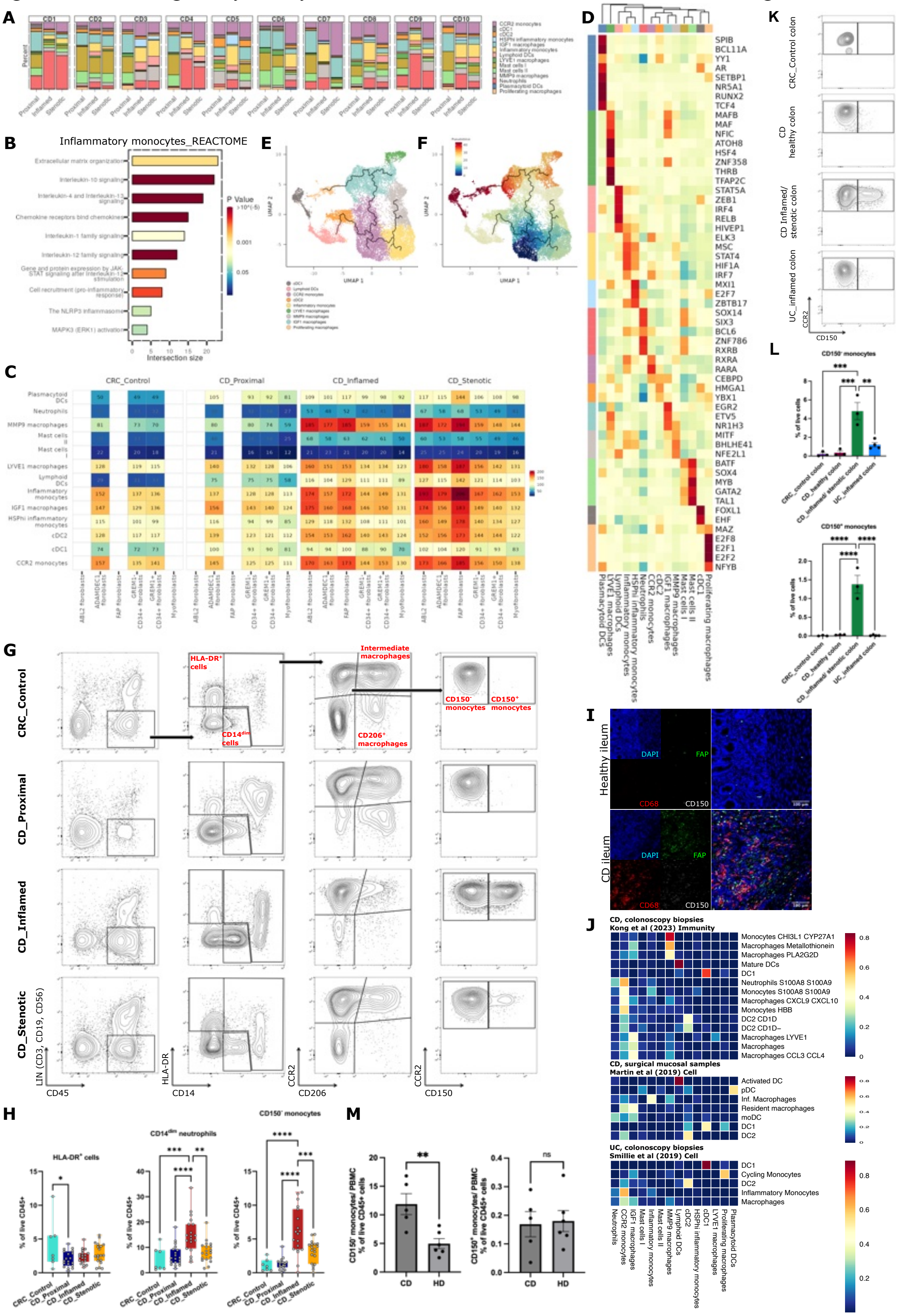

Figure S5. Gene expression of FAP fibroblasts and Inflammatory monocytes in inflamed and stenotic ileum of fibro-stenotic CD patients, related to Figure 5.

A

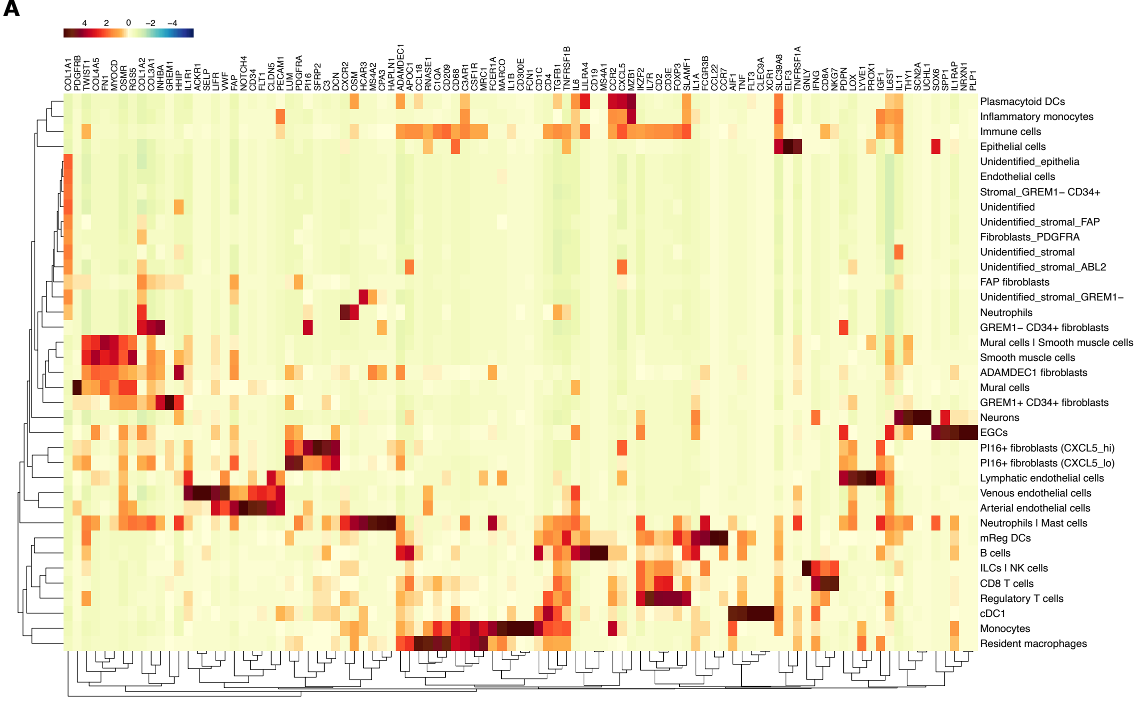

Figure S6. CD150⁺ monocytes-derived cytokines promote FAP fibroblast activation and extra-cellular matrix protein deposition, related to Figure 6.

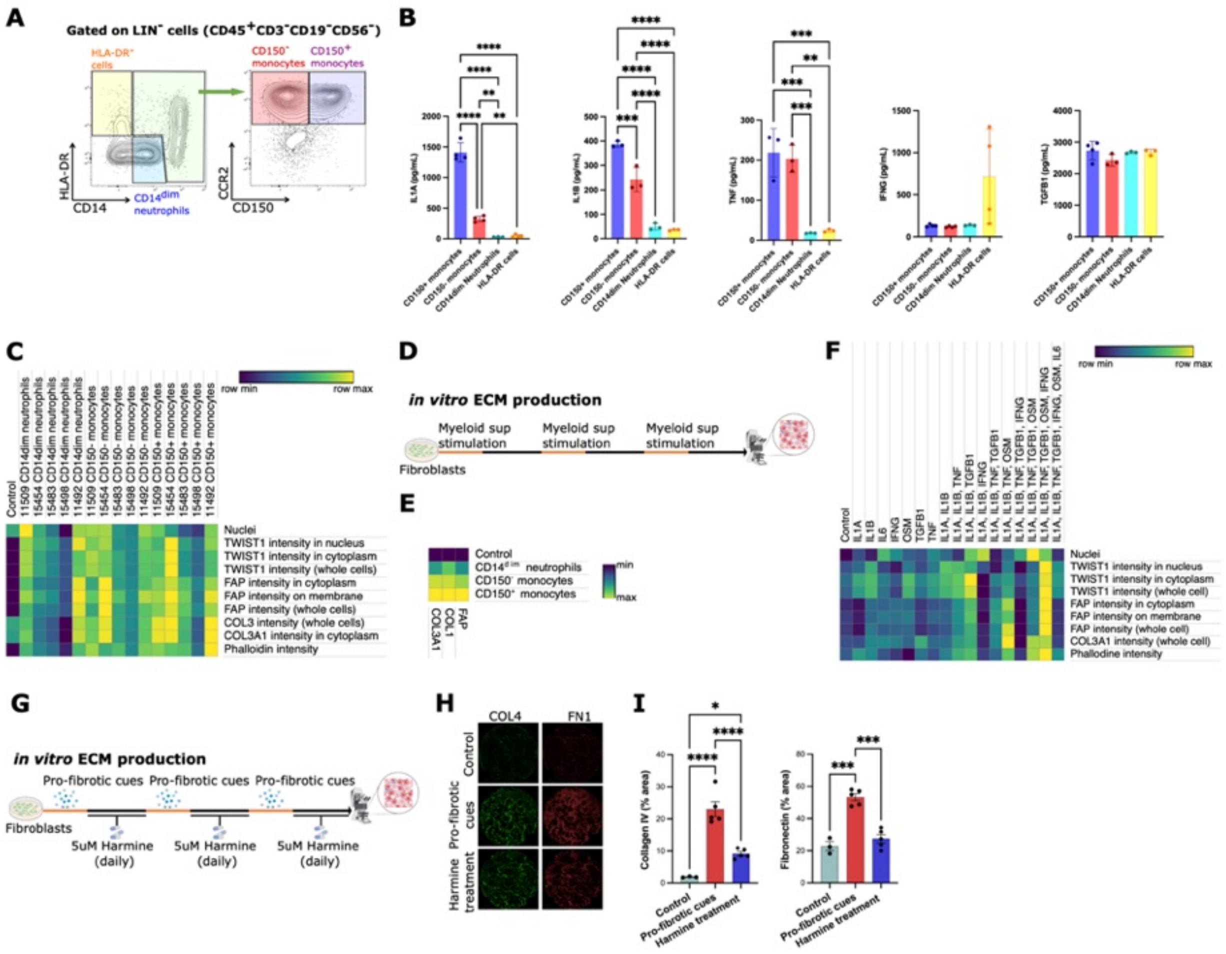

Figure S7. TWIST1 inhibition attenuates intestinal fibrosis.

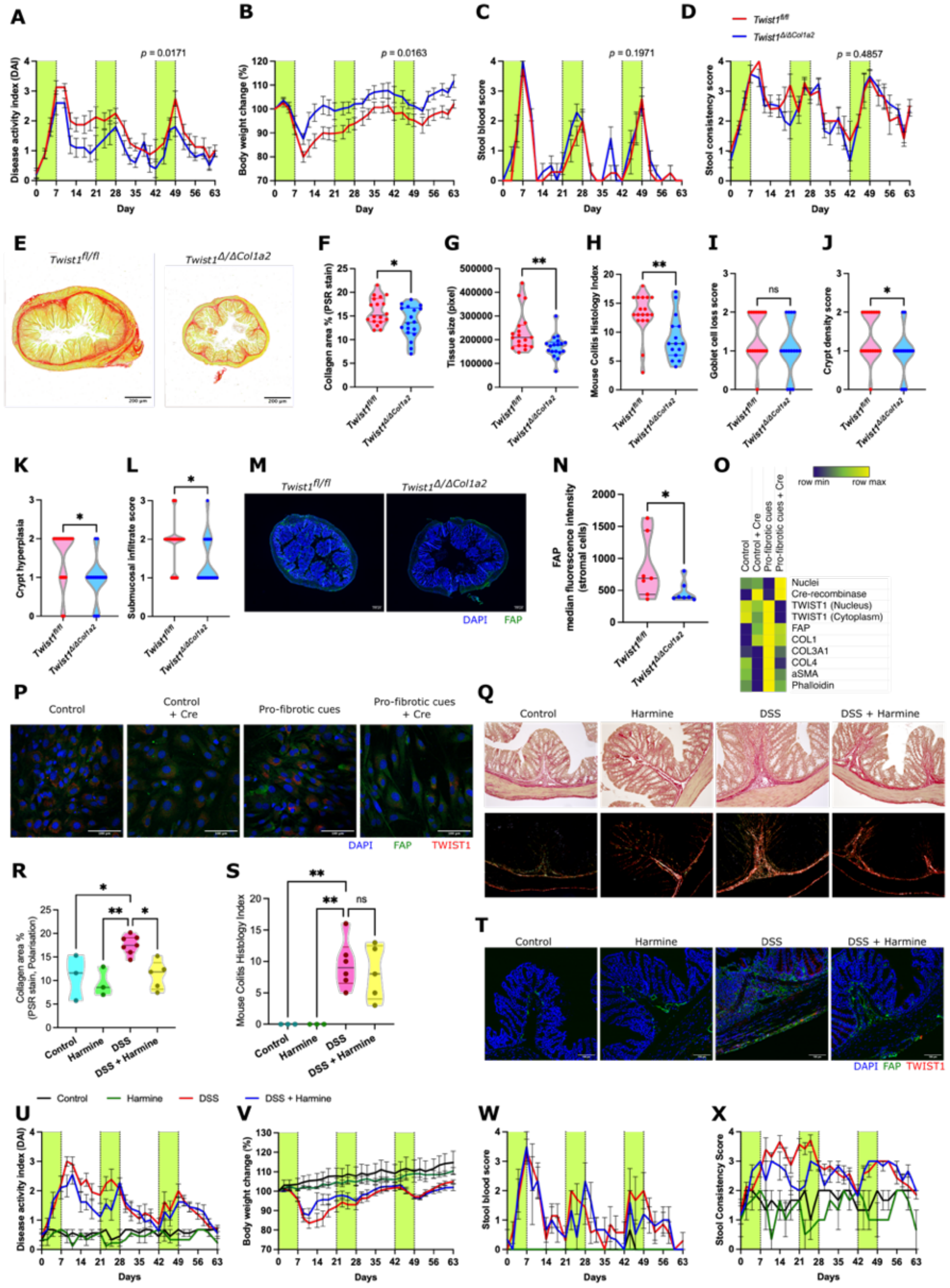

Figure S8. Previously published datasets using mucosal biopsies do not show consistent presence of FAP fibroblasts and inflammatory monocytes across IBD patients, related to Figure S2 and S4.

A

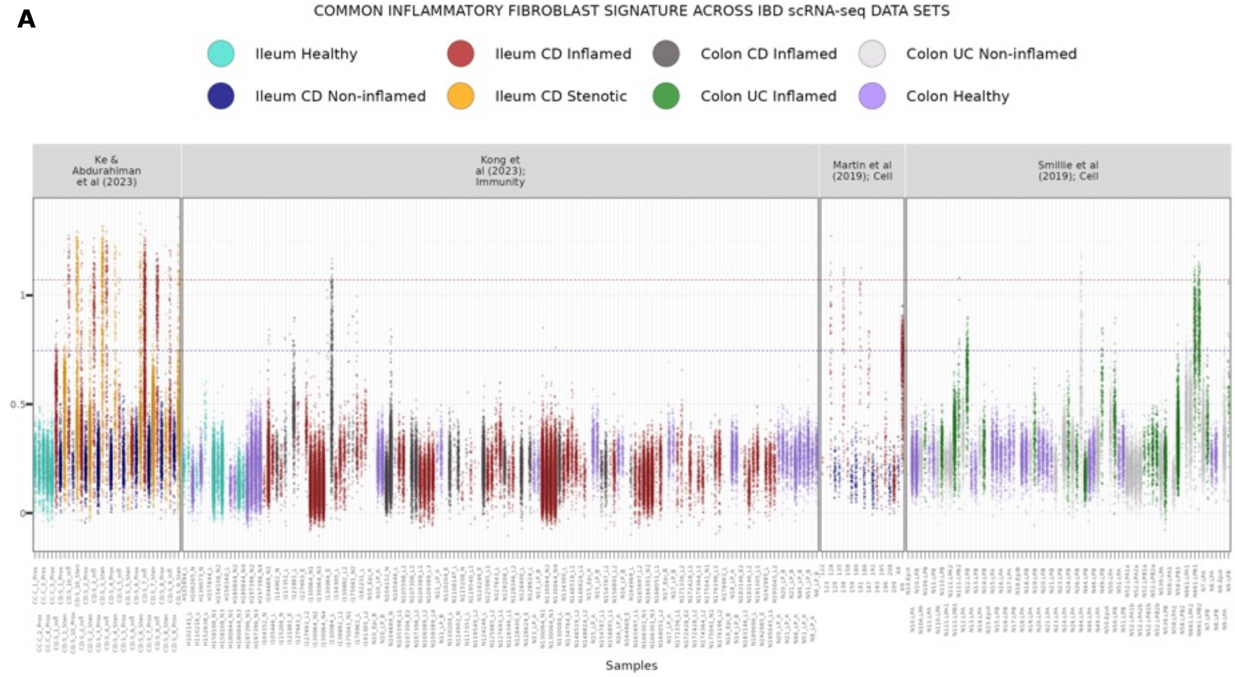

B

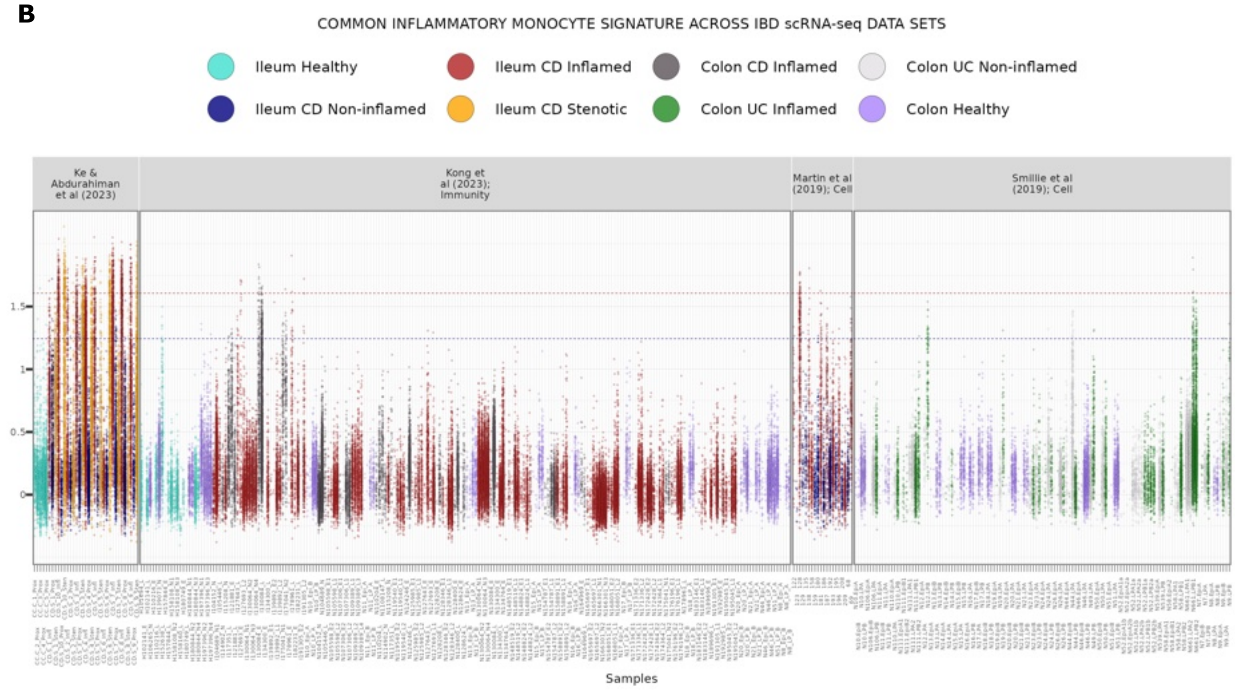

Supplement: Supplemental data [file jci-134-173835-s067.pdf]
